# Mussel-inspired immunomodulatory and osteoinductive dual-functional hydroxyapatite nanoplatform for promoting bone regeneration

**DOI:** 10.1186/s12951-024-02593-3

**Published:** 2024-06-08

**Authors:** Danlei Qin, Yifan Zhao, Rui Cheng, Yingyu Liu, Susu Guo, Lingxiang Sun, Yanqin Guo, Fengxiang Hao, Bin Zhao

**Affiliations:** 1 Shanxi Medical University School and Hospital of Stomatology, Taiyuan, Shanxi 030001 China; 2 Shanxi Province Key Laboratory of Oral Diseases Prevention and New Materials, Taiyuan, Shanxi 030001 China; 3 Department of Medical Imaging, Shanxi Medical University, Taiyuan, Shanxi 030001 China; 4 Department of Endocrinology, The Second Hospital of Shanxi Medical University, Taiyuan, Shanxi 030001 China; 5 Department of Ultrasound, The Second Hospital of Shanxi Medical University, Taiyuan, Shanxi 030001 China

**Keywords:** Mussel-inspired nanocomposites, Bone repair, Immunomodulation, Osteoinductivity

## Abstract

Simultaneously modulating the inflammatory microenvironment and promoting local bone regeneration is one of the main challenges in treating bone defects. In recent years, osteoimmunology has revealed that the immune system plays an essential regulatory role in bone regeneration and that macrophages are critical components. In this work, a mussel-inspired immunomodulatory and osteoinductive dual-functional hydroxyapatite nano platform (Gold/hydroxyapatite nanocomposites functionalized with polydopamine - PDA@Au-HA) is developed to accelerate bone tissues regeneration by regulating the immune microenvironment. PDA coating endows nanomaterials with the ability to scavenge reactive oxygen species (ROS) and anti-inflammatory properties, and it also exhibits an immunomodulatory ability to inhibit M1 macrophage polarization and activate M2 macrophage secretion of osteogenesis-related cytokines. Most importantly, this nano platform promotes the polarization of M2 macrophages and regulates the crosstalk between macrophages and pre-osteoblast cells to achieve bone regeneration. Au-HA can synergistically promote vascularized bone regeneration through sustained release of Ca and P particles and gold nanoparticles (NPs). This nano platform has a synergistic effect of good compatibility, scavenging of ROS, and anti-inflammatory and immunomodulatory capability to accelerate the bone repair process. Thus, our research offers a possible therapeutic approach by exploring PDA@Au-HA nanocomposites as a bifunctional platform for tissue regeneration.

**Supplementary Information:**

The online version contains supplementary material available at 10.1186/s12951-024-02593-3.

## Introduction

Bone regeneration is a complex process in which various specific cells work together, and involves multiple physiological processes such as osteoblast differentiation, vascularization, and immune regulation [1, 2]. Osteoimmunology also suggests that successful bone healing is based on carefully coordinated crosstalk between inflammatory and bone-forming cells [3]. Previous studies have focused on optimizing the osteogenic properties of biomaterials, while the innate immune response is easily overlooked and may lead to failure of bone repair [4]. Moreover, vascularity is also essential for bone homeostasis and repair [5, 6], and it has been shown that poor angiogenesis or inadequate blood supply can lead to delayed bone healing or impaired osseointegration [7]. Therefore, attention to immune microenvironment regulation and vascularization in the early stages of bone tissue repair remains of great scientific value.

Recent developments in osteoimmunology have demonstrated that the immune system-of which macrophages are critical components-plays a crucial regulatory function in bone regeneration [8, 9]. Similarly, skin wound healing is also a complex process, and macrophages can drive inflammatory responses and also promote wound repair. Macrophages exhibit great plasticity and diversity that can differentiate into different phenotypes in response to a variety of stimuli, usually with two polarized phenotypes, M1 and M2 [10]. To fight against infections and stimulate inflammation, classically activated M1 macrophages can secret various pro-inflammatory cytokines including interleukin (IL)-1β, tumor necrosis factor-alpha (TNF-α), and inducible nitric oxide synthase (iNOS) [11]. In contrast, the alternatively activated M2 macrophages can produce anti-inflammatory cytokines to alleviate inflammation and repair damaged tissues, such as IL-10, arginase-1 (Arg-1) [12, 13]. Furthermore, it has been documented that macrophages can secrete growth factors that directly promote the creation of new bone and tissue vascularization, including transforming growth factor beta (TGF-β), vascular endothelial growth factor (VEGF), and bone morphogenetic protein-2 (BMP-2) [14, 15]. Therefore, regulating the polarization of macrophages toward the major M2 phenotype can create an ideal immune microenvironment for promoting bone regeneration and wound healing. According to a study, calcium nervonate NPs can combine immunomodulation and osteoinduction to promote bone regeneration in a dual-functional therapeutic platform [16]. Another study used a bionic glycopeptide hydrogel-coated PCL/nHA scaffold to induce macrophage M2 polarization and M2 macrophage-bone marrow mesenchymal stem cells (BMSC) crosstalk to promote osteogenesis [17]. In conclusion, there is a need to find effective ways to modulate the immune microenvironment during the inflammatory phase to provide favorable conditions for bone regeneration and vascularization during the proliferative remodeling phase, which ultimately leads to good repair of hard and soft tissues [18].

In the fields of tissue engineering, biological technology, and biomedical sciences, nanoparticle-based biomaterials have garnered a lot of attention. Studies on the immune-modulating ability of nano-biomaterials and their consequent impact on angiogenesis and osteogenesis are extremely rare. HA is the most used biomaterial in bone tissue engineering, and its inorganic composition is similar to natural bone, with excellent biocompatibility and osteoinductivity [19, 20]. HA is rich in Ca and P ions, which may promote the formation of apatite crystals [21]. However, the ability of HA to promote bone regeneration is limited and further modification is needed to improve osteogenic capacity. Au NPs are considered prominent nanomaterials in the biomedical field. There is growing evidence that Au NPs are a suitable osteoinductive biomaterial for the engineering and regeneration of bone [22, 23]. The Wnt/β-linker protein signaling pathway can be triggered by internalizing a new Au-HA NP into BMSCs. Combining the excellence of these two materials, Au was loaded on HA to achieve synergistic promotion of bone regeneration [24]. Au NPs also modulate macrophage polarization to promote bone repair [25, 26]. Combining mesoporous silica nanoparticles (MSNs) with Au revealed that Au-MSNs were able to regulate macrophage phenotype and provide favorable conditions for bone tissue regeneration [27]. When biomaterials are implanted into the area of bone defects, they can cause local inflammatory responses, oxidative stress and ROS production, which can lead to poor bone repair and delayed healing. PDA is regarded as a potential antioxidant that accelerates wound healing by reducing oxidative stress by scavenging ROS and downregulating inflammatory mediators [14, 28–30]. PDA is synthesized from dopamine (DA) by electropolymerisation or solution oxidation or enzymatic oxidation [31]. Mussels produce adhesion proteins, mytilus foot protein 5, is rich in 3,4-dihydroxy-L-phenylalanine, from which the neurotransmitter DA is derived [32]. Moreover, PDA is rich in functional groups (catechol, amine, and imine) that can interact with amino groups in tissues, exhibiting good adhesion and hemostatic effects [33]. Recently, PDA coatings have extensive adhesion, high chemical reactivity, excellent biocompatibility, and strong photothermal properties, which have become a surface functionalization method for various materials [34]. PDA-based biomaterials can provide a localized regenerative microenvironment for hard and soft tissues healing. In this study, inspired by the phenomenon of mussel bonding, we introduce PDA coatings to Au-HA surfaces. Moreover, PDA has cell/tissue affinity to promote cell adhesion, mainly due to the interaction of the catechol group with the amino or sulfhydryl groups of the tissue.

Our goal in this work is to create a bone substitute that can actively control the immune microenvironment and encourage the regeneration of vascularized bone. We designed a mussel-inspired PDA@Au-HA nano platform that regulates the microenvironment throughout the wound healing process and ultimately promotes vascularized bone regeneration Scheme1). We have investigated the morphology, physical structure, and chemical composition of the PDA@Au-HA nano platform, which is comprised of HA, Au, and PDA coatings. When nanomaterials are implanted into the defect site, the host’s innate immune system is first activated to cause a local inflammatory response and excessive ROS production. PDA has a large number of catecholamine groups on the surface to scavenge ROS, sense oxidative stress in the traumatic area and modulate macrophage phenotype to down-regulate the pro-inflammatory factors, ultimately providing a favorable immune microenvironment for osteogenic differentiation. In the bone proliferation and reconstruction phase, widely recognized as a bioactive substance, HA affects not only the behavior of cells but also helps with adhesion, proliferation, and differentiation by forming a phosphate layer to adsorb protein molecules. PDA@Au-HA nanocomposites are capable of releasing Ca and P ions as well as Au NPs, and the multifactorial synergistic action promotes bone regeneration as well as blood vessel formation. Since bone immunomodulation cannot be well monitored in early in vivo, in this study we evaluated the effects of different nanomaterials on the local immune microenvironment during the inflammatory phase by further constructing a skin defect model. Therefore, mussel-inspired PDA@Au-HA nanocomposites provide an effective strategy for regenerative repair involving multiple systems.

**Sch. 1:**
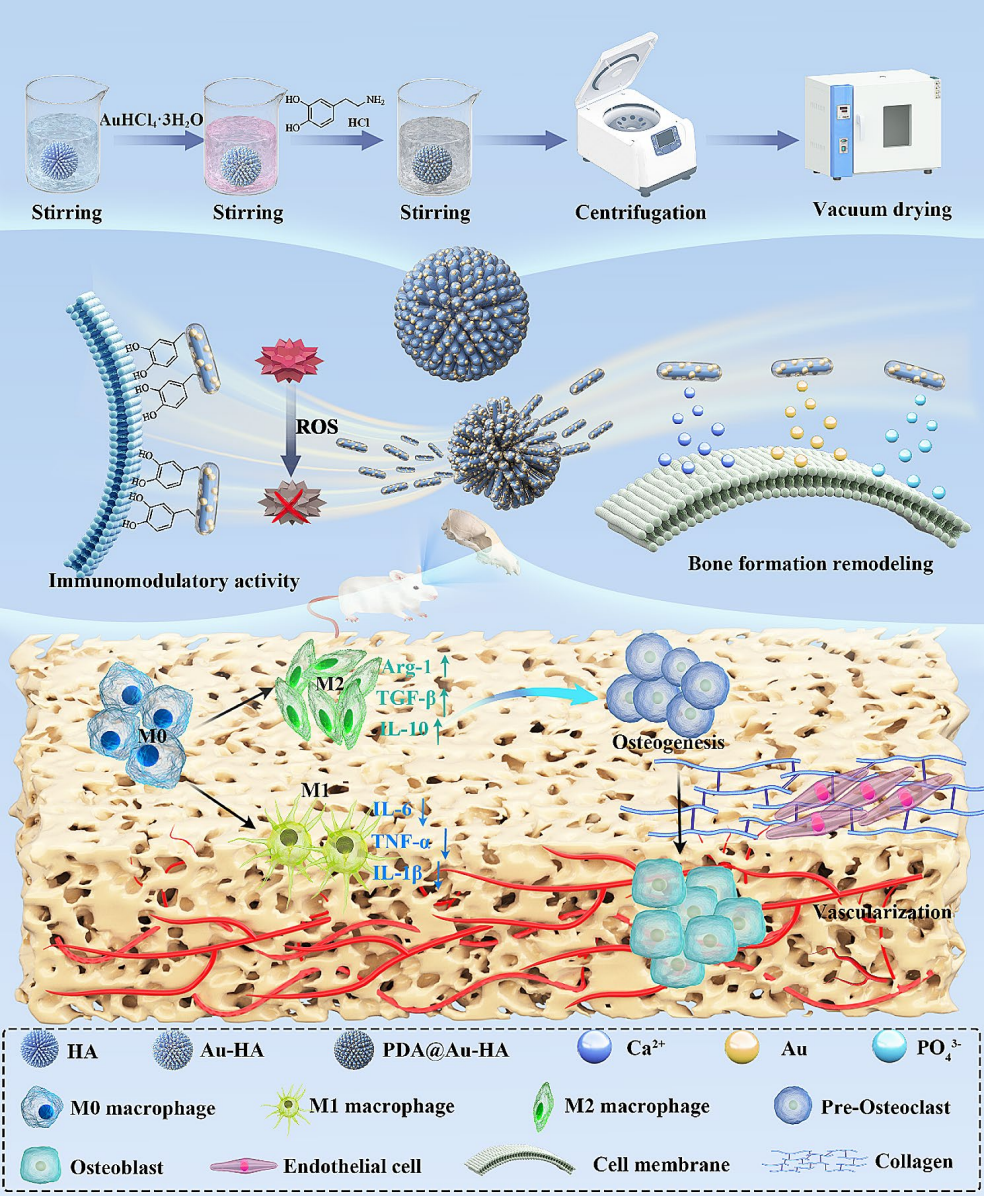
Schematic illustration of design of PDA@Au-HA NPs and their Anti-inflammatory immunomodulation and vascularized bone regeneration functions to promote the bone repair.

## Materials and methods

### Synthesis of PDA@Au-HA nanoparticles

We prepared the nanocomposites using a simple precipitation deposition method and synthesized them as follows. After dissolving 0.12 g of HAuCl_4_·3H_2_O (Macklin, China) in 90 mL of DI water and vigorously swirling in 0.1 M NaOH (Macklin, China), the PH was brought to 9. After that, 1.5 g of HA (Macklin, China) was added to the HAuCl_4_ aqueous solution, and NaOH was used to bring the pH down to 9. After two hours of stirring, the product was heated to 65 °C. For later usage, the precipitate was washed with DI water until it was free of chloride ions and it was then dried at 60 °C for an entire night. The samples were calcined at 500 °C ultimately for 3 h. 2 g of the prepared Au-HA was stirred in 1 mg/mL tris-buffer (Macklin, China) solution containing dopamine for 24 h (100 mL, 10 mM; pH 8.5). The sample was vacuum-dried at 40 °C after being cleaned three times with DI water.

### Physicochemical characterizations of the nanoparticles

The samples’ structures were examined using transmission electron microscopy.

(TEM, Tecnai G20 electron microscope, FEI, USA). Energy Dispersive Spectroscopy (EDS) and X-ray photoelectron spectroscopy (XPS, ESCALAB 250Xi, Thermo Scientific, USA) were used to analyze the chemical element components of NPs. Using Fourier transform infrared spectroscopy (FTIR) at a wavenumber ranging from 500 to 4000 cm^− 1^, the functional group change of the samples was assessed. Inductively coupled plasma atomic emission spectrometry (ICP–AES) was used to determine the contents of Au, Ca and P at 1, 3, 5, 7 and 14 days. The PDA@Au-HA sample was dried and weighed, and 50 mg of sample was added to 25 mL (m/v = 2 mg/mL) of PBS solution. After incubation in a 37 ◦C constant-temperature water bath shaker for 1, 3, 5, 7, and 14 days, and the superatant was removed by centrifugation. There were 3 parallel samples in each group. Since PBS contains phosphate and the concentration C0 of P in PBS is measured at the same time, C0 needs to be subtracted when calculating the release amount of P ions.

### Cell culture

The China Center for Type Culture Collection (CCTCC) provided the mouse pre-osteoblastic cell line MC3T3-E1, the human umbilical vein endothelial cells (HUVECs), and the mouse-derived macrophage cell line RAW 264.7 cells. MC3T3-E1 cells were cultured in α-modified minimum essential medium (α-MEM, BOSTER, USA), whereas RAW 264.7 cells and HUVECs were grown in high glucose Dulbecco’s modified Eagle’s media (DMEM, Gibco, USA). Under conventional growth conditions (37 °C, 5% CO_2_, and 95% relative humidity), 10% fetal bovine serum (Cellmax, China) and 1% penicillin-streptomycin solution (Meilunbio, China) were added to both types of media. The cells were passaged at about 80% and were used in experiments at passages 3–5.

### Biocompatibility and cytotoxicity research of the PDA@Au-HA

Cell viability and proliferation were detected with the CCK-8 kit (Solarbio, China). Briefly, RAW264.7 and MC3T3-E1 cells were seeded in 96-well plates with a cell density of 5 × 10^3^ cells per well overnight. Then, these cells were treated with HA, Au-HA, and PDA@Au-HA at different concentrations (25, 50, 75, 100, 150, or 200 µg/mL). There were six parallels for each group. After culturing for 24 h, the optical density (OD) values were measured at 450 nm using a microplate reader. To further evaluate the proliferation of the samples, MC3T3-E1 and HUVECs cells were cultured with the NPs for 1, 3, and 5 days in the plates. Using a live/dead staining kit (Beyotime, China) and a fluorescence microscope (Olympus, Japan), cell survival was identified.

Cytoskeleton Observation. To find out how the NPs affected the cell shape, fluorescence staining was used. MC3T3-E1 and HUVECs cells were seeded at a density of 5 × 10^4^ cells per well into 24-well cell slides. After being co-cultured with the NPs for a day, they were fixed in 4% paraformaldehyde (Meilunbio, China) for 30 min and then penetrated with 0.5% Triton X-100 (Solarbio, China) for 10 min. The nuclei were stained with 4′,6-diamidino-2-phenylindole (DAPI) (Solarbio, China), and the actin filaments were stained with TRITC-Phalloidin (Solarbio, China) following three PBS washes. The cytoskeleton was observed by confocal laser microscope(CLSM, Olympus, Japan). (DAPI Ex/Em:358/461 nm, TRITC Phalloidin Ex/Em:540–546/565–575 nm).

Hemolysis assay. A 1 mL murine blood sample stabilized with ethylene diamine tetra-acetic acid was centrifuged for 10 min at 3000 rpm. Subsequently, 0.02 mL of red blood cell solution was removed and combined with 1 mL of NPs at varying concentrations (20, 50, 75, 100, 150, and 200 µg/mL), 1 mL of ddH_2_O (positive control), and 1 mL of PBS (negative control). All samples were then gently vortexed and allowed to stand at ambient temperature for four hours. The resulting dispersions were then centrifuged for five minutes at a speed of 12,000 rpm, and observe the hemolysis situation.

Biosecurity study. Used different nanomaterials to intervene in rats and observe changes in the wound site. At the end of the observation, important organs (heart, liver, spleen, lungs, kidneys) from each group were taken and subjected to hematoxylin and eosin (H&E) staining for histopathological examination.

### ROS scavenging research in vitro

A reactive oxygen species assay kit (2′ ,7′-dichloro fluorescein diacetate, DCFH-DA, Beyotime, China) was used to test the cellular ROS scavenging activity. RAW 264.7 cells (5 × 10^5^ cells per well) were seeded in a 6-well plate and treated with HA, Au-HA, and PDA@Au-HA with or without lipopolysaccharide (LPS, Solarbio, China) for 24 h. The DCFH-DA (10 µM) probe was first added to each well. After 30 min of stimulation (37 °C, 5%CO_2_), the ROS levels were measured using an orthogonal fluorescence microscope (Olympus, Japan).

### Anti-inflammation and macrophages polarization research in vitro

Initially, LPS was used as the trigger for the status of inflammation. In a 6-well plate, RAW 264.7 cells were seeded at a density of 5 × 10^5^ cells/well and cultivated for a day. Subsequently, LPS (1 µg/mL) was added to activate the cells for 12 h to mimic the inflammation in vitro before various NPs were added. Following a 24-hour incubation period, the cells were separated by centrifugation, subjected to two rounds of cold PBS washing, and stained using anti-iNOS antibodies (Biolegend, USA) labeled with APC, anti-F4/80 antibodies (Biolegend, USA) labeled with FITC, and anti-CD206 antibodies (Biolegend, USA) labeled with PE. Utilizing flow cytometry (Agilent, USA), the proportion of polarized macrophages was determined. Using qPCR, the mRNA expression levels of IL-1β, IL-6, TNF-α, IL-10, TGF-β, and Arg-1 were used to assess the M1/M2 phenotypic inflammatory response of RAW 264.7.

### Osteogenic differentiation MC3T3-E1 cultured with conditioned medium in vitro

Collection and preparation of conditioned medium (CM). RAW 264.7 cells were cultured in a growth medium supplemented with HA, Au-HA, and PDA@Au-HA for 3 days. The culture supernatants were collected and diluted with α-MEM containing 10% FBS at a ratio of 1:1 before use.

Alkaline Phosphatase (ALP) Analysis. MC3T3-E1 cells were seeded in a cell culture dish with 1.5 × 10^5^ cells per dish. The macrophage-CM (HA, Au-HA, and PDA@Au-HA) was refreshed every 3 days. ALP staining was performed using a BCIP/NBT alkaline phosphatase color development kit (LEAGENE, China) on the 7th day, and photographic images were captured via a stereomicroscope (Olympus, Japan).

Alizarin Red S (ARS) Staining. The MC3T3-E1 cells were cultured with different macrophage-CM for 14 days in the culture dish. The cells were preserved for 20 min at room temperature using 4% paraformaldehyde. After three PBS washes, the cells were stained for ten minutes with 1% (w/v) Alizarin Red S (pH = 4.2) (Haixing, China). We used an Olympus IX73 inverted fluorescence microscope to view the samples stained with ARS.

### Angiogenesis-related research in vitro

HUVECs were planted at a density of 4 × 10^5^ cells per well in a six-well plate for wound healing tests. Following complete cell fusion, a sterile 200 µL pipette tip was used to produce a cell-free “scar” in the center of the well. The shed cells were then cleaned and eliminated using PBS. The well was added with the culture media containing 200 µg/mL of various NPs. At 0 and 12 h following treatment, the images were captured using inverted optical microscopy (Leica, Germany), and Image J software was used for analysis.

For the Transwell migration assay, different NPs (200 µg/mL) were added to the lower chamber containing high-sugar DMEM medium. Next, 200 µL of the cell suspension (2 × 10^5^/mL) was put into the Transwell’s upper chamber. Using a cotton swab, the residual cells in the Transwell insert’s upper chamber were removed after 24 h. The cells in the lower side were fixed with 4% paraformaldehyde and stained with 0.1% crystal violet, then, washed with PBS to remove the excessive staining solution. Optical microscopy was used to take pictures of each group. For quantitative analysis, the number of migrated cells in different groups was analyzed by Image J.

Tube Formation Assay: To investigate the effect of the NPs on angiogenesis, HUVECs were treated with the extracts of different NPs (HA, Au-HA and PDA@Au-HA) for 24 h. Then, the cells (2 × 10^4^/well) were seeded in 96-well plates precoated with Matrigel (Corning, NY, USA). After 6 h, three random fields were captured with an inverted microscope (Olympus, Japan), and the images were analyzed by Image J software.

### Real-time quantitative PCR (RT-qPCR)

After MC3T3-E1 cells were cultured with different macrophage-CM for 7 and 14 days. LPS induced the inflammatory response of RAW264.7 cells at a concentration of 1 µg/mL. After RAW 264.7 cells were cultured with 200 µg/ml HA, Au-HA, and PDA@Au-HA for 24 h. Then, the total RNA was extracted using an M5 Universal RNA Mini Kit (Mei5bio, China), followed by reverse transcription into cDNA using an M5 Super qPCR RT Kit (Mei5bio, China). Finally, a gene quantitative system was used to perform RT-PCR (Mei5bio, China) to measure the expression of TNF-α, IL-1β, IL-6, TGF-β, IL-10, Arg-1, Runt-related transcription factor 2 (Runx2), osteopontin (OPN), bone morphogenetic protein (BMP-2), ALP, osteocalcin (OCN), GAPDH. The results were normalized to GAPDH expression levels and analyzed using the comparative 2^−ΔΔCt^ method.

### Western blot (WB)

The MC3T3-E1 cells were cultured with different macrophages CM for 14 days. LPS induced the inflammatory response of RAW264.7 cells at a concentration of 1 µg/mL. After RAW 264.7 cells were cultured with 200 µg/ml HA, Au-HA, or PDA@Au-HA for 24 h. These cells were lysed using the RIPA Lysis Buffer (BOSTER, China) containing 1 mM PMSF (BOSTER, China) for 0.5 h to extract total protein. The protein concentration was determined using the BCA kit (BOSTER, China). The primary antibodies were produced by HuaBio (diluted 1000 times for iNOS, CD206, Runx2, OPN, ALP), with internal control of GAPDH (Proteintech, China). A BIO-RAD chemiluminescent imaging system (Bio-Rad chemi doc XRS+, USA) was selected to apply for observing the proteins after 1 h incubation of the membranes with a secondary antibody. The semi-quantitative analysis of proteins was carried out using densitometry (Image J), and all results were normalized to GAPDH.

### Immunofluorescence staining (IF)

The MC3T3-E1 culture with different macrophages CM for 7 days. HUVECs and LPS-activated RAW264.7 cells were culture with 200 µg/ml of HA, Au-HA, PDA@Au-HA for the corresponding days. Then, these cells were fixed with 4% paraformaldehyde for 30 min. After that, the cells were blocked for 30 min at room temperature with 1% bovine serum albumin after being permeabilized for 15 min with 0.1% Triton X-100. The cells were then incubated with primary antibodies (diluted 200 times for iNOS, CD206, IL-6, Arg-1, Runx2, BMP-2, ALP, OPN, VEGF) at 4 ◦C overnight. The appropriate secondary antibodies (diluted 200 times) were added and incubated for 1 h after three PBS washes. F-actin and nuclei were stained with 100 nM TRITC-phalloidin and 1 µg/mL DAPI, respectively, following three PBS washes. Each group was imaged using a laser confocal microscope (Olympus, Japan). The fluorescence intensity was quantified using Image J software.

### In vivo experiments

The Ethics Committee of Shanxi Medical University, School of Stomatology gave its approval to all animal research. Sprague-Dawley (SD) rats (male, 7 weeks old, weighing approximately 200 g) were purchased from the Animal Experiment Centre of Shanxi Medical University. A 21 °C, 50% humidity, and 12-hour light/dark cycle was maintained in the housing of male SD rats. Then, a rat cranial defect model was constructed to explore bone remodeling. 18 SD rats were randomly divided into 3 groups on average (control + HA group, control + Au-HA group, control + PDA@Au-AH group, 6 SD rats/group). A 5-mm round bone defect was created in the parietal bone on each side of the sagittal suture of rats, and different materials were placed in the defect on the right side, while the left side was used as a blank control group. The rats were euthanized after 8 weeks of rearing. Cranial samples were fixed and subjected to H&E staining, Masson trichrome staining, Immunohistochemical (IHC) staining, and Micro-CT. Finally, quantitative analyses were performed using Image J.

For this animal wound healing investigation, six-week-old SD rats weighing between 150 and 200 g were utilized. A total of 24 SD rats were randomly divided into four groups, with 6 rats in each group (control group, HA, Au-HA, and PDA@Au-HA group). On day 0, all rats were administered anesthesia and shaved, and a round full thickness of skin defect (diameter of 2 cm) was created on the dorsal side of each rat. The rats were treated with different NPs (200μg/ml) every day. The wound closure rate was evaluated by taking digital photos on days 0, 3, 7 and 14 and further calculated using Image J software. On days 3, 7, and 14, skin tissue samples were taken and preserved for 24 h in 4% formaldehyde buffer. Following dehydration using xylene and ethanol solutions, the samples were embedded in paraffin and sliced. Wound healing was assessed by H&E and Masson staining on days 7 and 14. IHC staining with IL-6, TNF-α, iNOS, and CD206 was performed on day 3 to assess wound inflammation. IHC staining with VEGF and platelet endothelial cell adhesion molecule-1 (CD31) was performed at the proliferation phase to assess vascularization of the wound.

### Statistical analysis

Statistical analysis on all experimental data of the study was performed, and the results are expressed as mean ± standard deviation (SD). One-way analysis of variance (ANOVA) (Tukey’s post-hoc test) was used for comparison among multiple groups, and statistical significance was considered as P value < 0.05.

### Result and discussion

#### Characterization of the PDA@Au-HA NPs

**Fig. 1:**
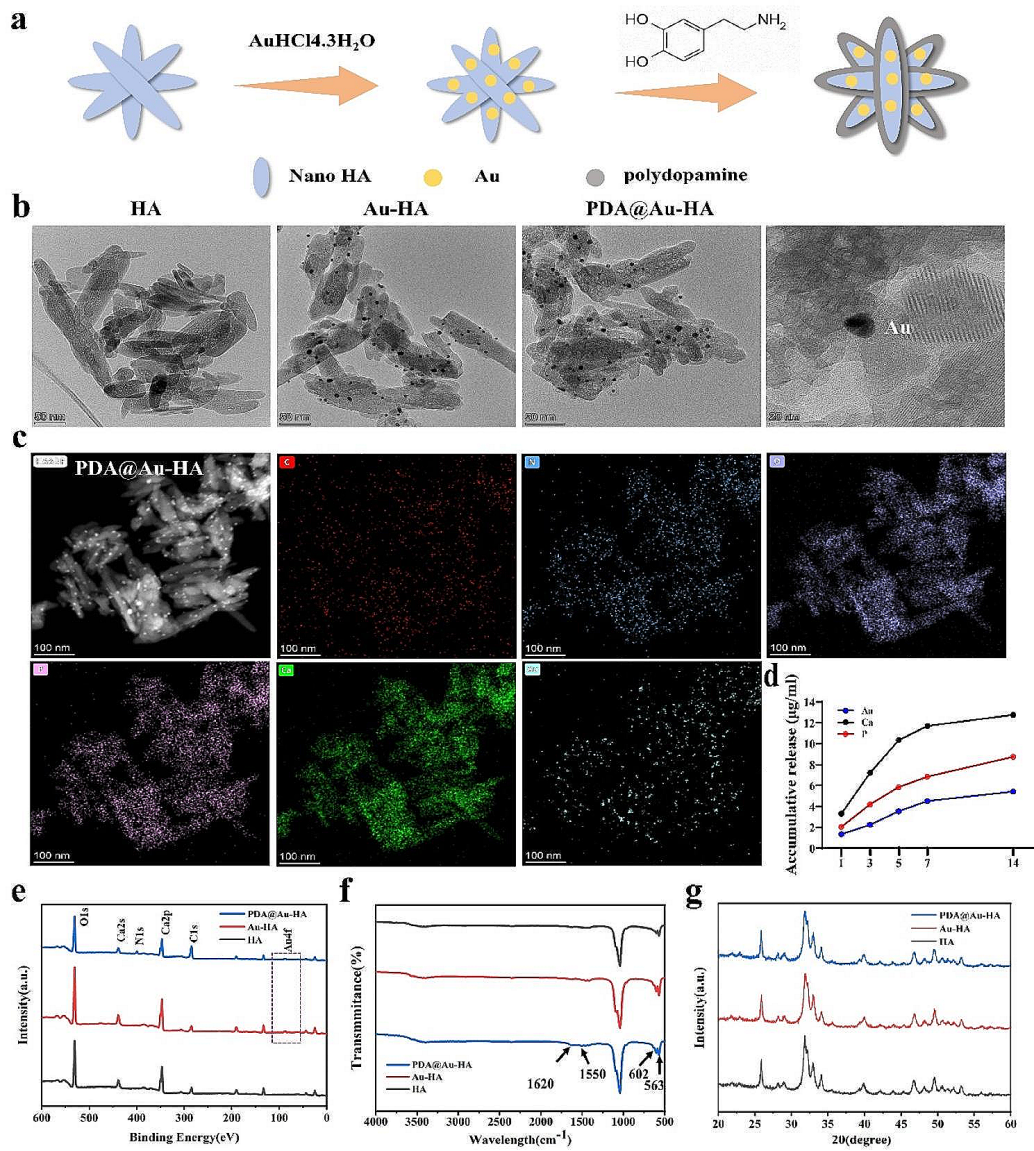
Characterization of HA, Au-HA and PDA@Au-HA NPs. (**a**) Nanocomposites synthesis flowchart. (**b**) TEM image of HA, Au-HA and PDA@Au-HA (scale bar:50 nm). (**c**) EDS image of PDA@Au-HA (scale bar:100 nm). (**d**) Ionic release behavior. (**e**) XPS pattern of NPs. (**f**) FT-IR spectra. (**g**) XRD analysis of the NPs (JCPDS: 09-0432)

We prepared PDA@Au-HA NPs using the deposition precipitation method and Tris-alkali buffer method. Figure 1a shows the schematic illustration of the PDA@Au-HA synthesis. The shape of the HA NPs was visible in the TEM images, and the Au NPs were evenly dispersed throughout each HA NPs. The PDA coating had no discernible effect on the Au-HA nanorods’ form **(**Fig. 1b**)**. EDS mapping demonstrated that the characteristic elemental C, N, Au in PDA@Au-HA was observed, indicating that Au and PDA was successfully loaded **(**Fig. 1c**)**. Then the release of Ca, P and Au from the nanocomplexes was studied. The contents of Ca, P, and Au elements in PBS gradually increased with time within 14 days, and increased faster in the first 5 days of the experiment, Then, the release rate basically reached an equilibrium on the 14th day **(**Fig. 1d**)**. Additionally, As seen in Fig. 1e, the N 1s signal is seen in the PDA@Au-HA, and an increase in O 1s peak intensity indicates that the PDA coating was successful. PDA coating may mask some of the Au 4f signals displayed by Au-HA. FTIR spectra tests of the NPs were performed. The characteristic bands at 1620 cm^− 1^ and 1550 cm^− 1^ originate from amide I and II, suggesting excellent adhesion between HA and PDA coating, while the bands at 563 cm^− 1^ and 601 cm^− 1^ matched to the P–O bending of the phosphate group arising from -PO4 in HA (Fig. 1f). In Fig. 1g, the XRD patterns are displayed. The diffraction peaks and planes correspond to the expected peaks of HA (JCPDS: 09-0432) [35]. The 2 theta angles of 25.9° correspond to the (002) crystal plane of HA and the three diffraction peaks at 31°–33° correspond to the (211), (112), and (300) crystal plane of HA [36].

### Biocompatibility evaluation of the PDA@Au-HA NPs

**Fig. 2:**
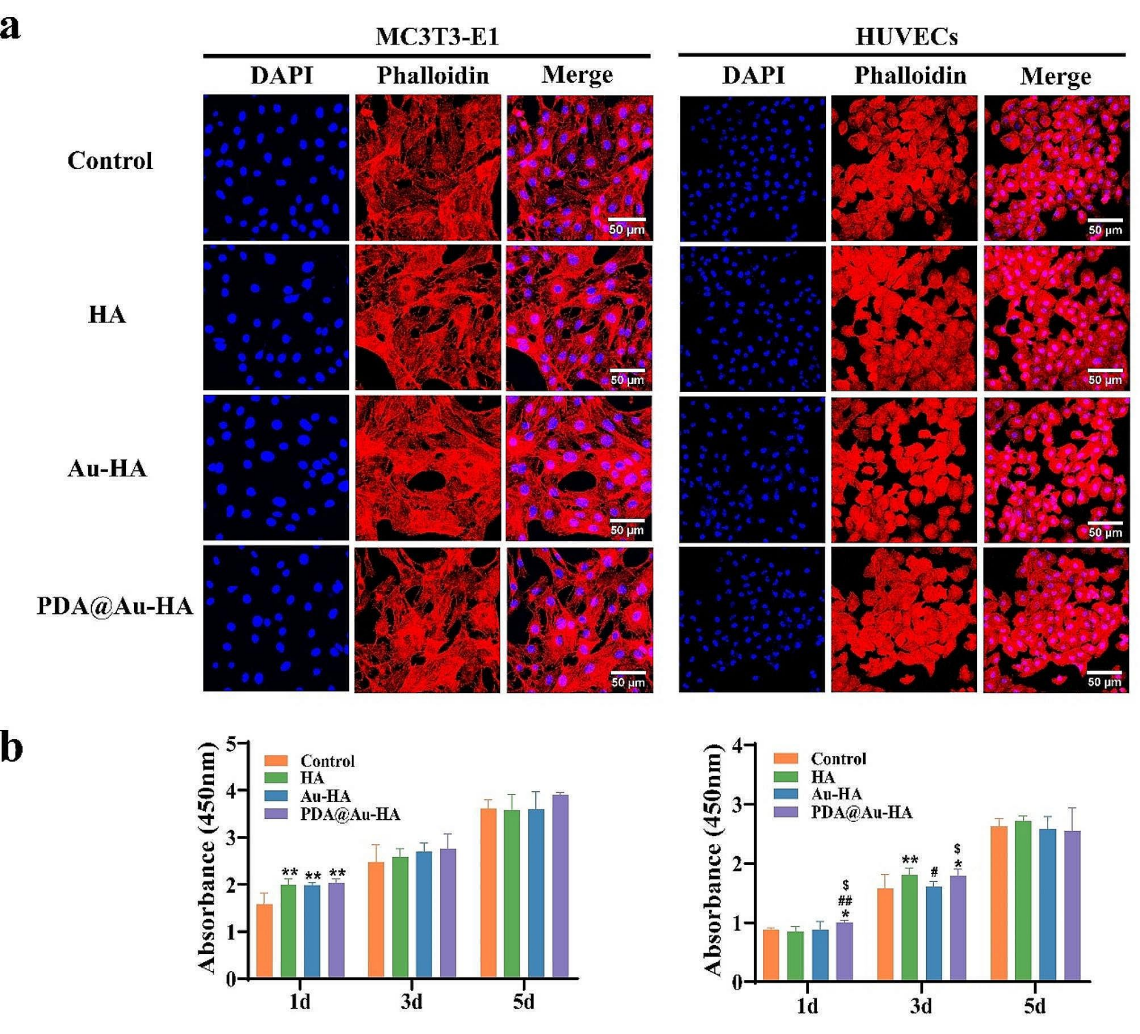
Evaluation the cytocompatibility of the HA, Au-HA and PDA@Au-AH NPs. (**a**) Cell adhesion of MC3T3-E1 and HUVECs after culturing for 24 h(scale bar:50 μm); (**b**) Cell proliferation of MC3T3-E1 and HUVECs after culturing for 1d, 3d and 5d. (*n* = 3. *p < 0.05 and **p < 0.01 indicate significant difference compared to the control group; ^#^*p* < 0.05 and ^##^*p* < 0.01 indicate significant difference compared to the HA group. ^$^*p* < 0.05 indicate significant difference compared to the Au-HA group)

Good biocompatibility is a necessary requirement for the clinical application of nanomaterials. The biocompatibility of the three NPs (HA, Au-HA, and PDA@Au-HA) was assessed by hemolysis test, cytoskeleton staining, the Cell Counting Kit-8 (CCK-8), and live/dead staining assays. In this study, firstly, we co-cultured RAW264.7 and MC3T3-E1 with different concentrations of NPs, respectively. Given that all of the NPs in the 0–200 µg/mL range demonstrated high biocompatibility according to the CCK-8 results in Fig. S1, the concentration of 200 µg/mL for HA, Au-HA, and PDA@Au-HA NPs was selected for more research. Then, cytoskeletal and morphological was observed through fluorescein TRITC-Phalloidin and DAPI staining. After adding different NPs to intervene in the MC3T3-E1 and HUVECs, the results shown that there is any difference in cytoskeleton and morphology among all groups. All cells can adhere and spread well on the bottom of the culture plate. Therefore, we believe that these NPs have good cell compatibility (Fig. 2a). For 1, 3, or 5 days, the MC3T3-E1 and HUVECs were cultivated with 200 µg/mLof HA, Au-HA, or PDA@Au-HA NPs. When cells co-cultured with the various NPs, the CCK-8 assay showed that cell viability in comparison to the control group remained unaltered, indicating the absence of harmful effects. After three and five days of culture, both MC3T3-E1 and HUVECs absorbance increased in all groups, indicating that the formulated NPs could promote cell proliferation **(**Fig. 2b**)**. The cell attachment seen in this study may indicate increased cell proliferation, as previous research has shown a positive association between the two. HA can form a phosphate layer to adsorb protein molecules, promoting cell adhesion and proliferation [37]. And various groups on the surface of PDA can interact with cells to promote cell proliferation [32].

We further used live/dead staining to demonstrate the biocompatibility of these NPs. The live/dead staining results in Fig. S2 highlight the good cytocompatibility of these three NPs further, as the numbers of dead cells (red) in MC3T3-E1 and HUVECs were negligible in all groups and the cell (green) survival rates were over 100%. Since wound dressings invariably come into touch with blood, a hemolysis experiment was then carried out to further assess the hemocompatibility of the NPs. Incubation of HA, Au-HA, and PDA@Au-HA with blood cells did not result in any apparent hemolysis compared with the water group, indicating that the blood compatibility of these NPs was good (Fig. S3). Taken together, these results indicate that PDA@Au-HA nanocomposites are biocompatible, non-hemolytic, supporting cell adhesion, growth, proliferation. Toxicity studies were conducted to assess the safety of HA, Au-HA, and PDA@Au-HA NPs after in vivo administration. The animals were in good condition because there were no odd reactions, infections, or movement impairments at the administration site or anywhere else topically. Major organs (heart, liver, spleen, lung, and kidney) stained with H&E in the HA, Au-HA, and PDA@Au-HA groups did not exhibit any overt toxicity or damage as compared to the control group (Fig. S4).

### Anti-inflammatory and immunomodulatory properties of PDA@Au-HA NPs

**Fig. 3:**
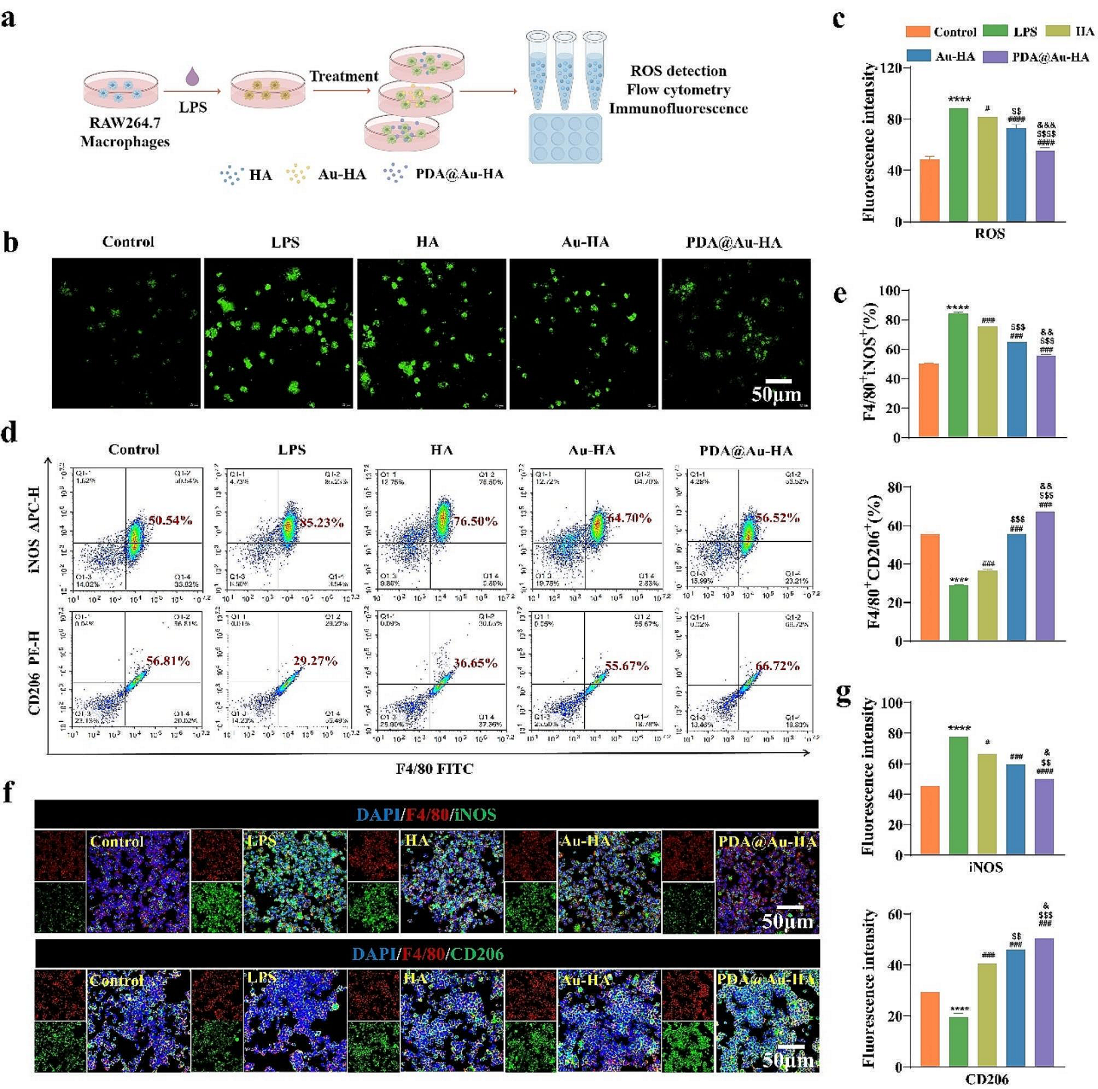
ROS scavenging and macrophage polarization property of PDA@Au-HA NPs. RAW264.7 cells were cultured with indicated groups. (control, LPS, HA, Au-HA and PDA@Au-HA. (**a**) In vitro inflammation modelling was drawn by Figdraw. (**b**) Intracellular ROS levels detection. Fluorescence images of intracellular ROS of RAW254.7 onto different samples, staining with DCFH-DA (scale bar:50 μm). (**c**) Statistical analysis of mean fluorescence intensity of ROS. (**d**) Flow cytometry analysis of the M1-like subset (F4/80^+^iNOS^+^) and M2-like subset (F4/80^+^CD206^+^). (**e**) Statistical analysis of the percent of M1 (F4/80^+^iNOS^+^) or M2 (F4/80^+^CD206^+^). (**f**) Representative IF images for iNOS (green) and CD206 (green) in macrophages cultured with different NPs, Nucleus (blue) (scale bar:50 μm). (**g**) Mean iNOS and CD206 fluorescence intensity. (*n* = 3. ****p < 0.0001 indicate significant difference compared to the control group; ^#^*p* < 0.05, ^###^*p* < 0.001 and ^####^*p* < 0.0001 indicate significant difference compared to the LPS group. ^$$^*p* < 0.01, ^$$$^*p* < 0.001 and ^$$$$^*p* < 0.0001 indicate significant difference compared to the HA group. ^&^*p* < 0.05, ^&&^*p* < 0.01 and ^&&&^*p* < 0.001 indicate significant difference compared to the Au-HA group)

In the present study, to investigate the immunoregulatory effect of PDA@Au-HA via macrophage polarization, an LPS-triggered inflammatory microenvironment was successfully constructed before the PDA@Au-HA treatment (Fig. 3a). Oxidative stress is an integral part after tissue damage. Excessive production of ROS accelerates the local inflammatory response and severely hinders hard and soft tissue regeneration [38]. A PDA@Au-HA has a large number of catechol antioxidant groups on its surface. First, the intracellular ROS scavenging capacity of RAW 264.7 cells in the treatment groups with oxidative stress damage was assessed using the DCFH-DA probe to illustrate the cellular ROS-scavenging property of the PDA@Au-HA NPs. Higher DCFH-DA staining was observed in the LPS group, suggesting that cellular oxidative stress was successfully induced. Not unexpectedly, the PDA@Au-HA group exhibited the lowest fluorescence signal, to a similar level as those in cells that were not exposed to LPS (control group) (Fig. 3b-c).

The host immune response is triggered by the implantation of biomaterials, and this is typically demonstrated by the macrophage phenotype. M1 macrophages promote inflammatory responses, whereas M2 accelerates tissue repair and bone regeneration [39]. Therefore, macrophage polarization is an attractive target to leverage for controlling inflammation and acquiring immune homeostasis for tissue reconstruction. In the present study, Flow cytometry was used to measure the conversion levels of macrophages in each group after the M1 and M2 phenotypes of macrophages were labeled with iNOS and CD206, respectively. As shown in Fig. 3d, the proportions of M1 macrophages on different NPs showed the following trend: PDA@Au-HA (56.52%) < Au-HA (64.70%) < HA (76.50%). Compared with those of the Au-HA, HA group, the lower iNOS-positive cells in the PDA@Au-HA group (56.52%) demonstrated the superior M1-type macrophage suppression effect of novel-developed nanocomposites. For the expression of CD206, the proportions of M2 macrophages on different nanomaterials showed the following trend: PDA@Au-HA (66.72%) > Au-HA (55.67%) > HA (36.65%). The higher percentage of CD206-positive cells in PDA@Au-HA groups indicated the prominent effect of nanocomposite on M2 macrophage polarization. The proportion of M1-like macrophages (F4/80^+^/iNOS^+^) in the PDA@Au-HA treatment groups was significantly lower than in the group stimulated by LPS, and the proportion of M2-like macrophages (F4/80^+^/CD206^+^) was increased **(**Fig. 3e**)**. IF labeling was used to visualize CD206 (the M2 marker) and iNOS (the M1 marker) under inflammatory circumstances (LPS stimulation) to further validate the polarization status of macrophages. IF staining results showed that iNOS intensity in the PDA@Au-HA group was significantly lower than those in the HA and Au-HA groups. In contrast, the CD206 intensity in the PDA@Au-HA group was the highest in all groups (Fig. 3f-g). In line with the flow cytometry study, the PDA@Au-HA groups showed increased M2 activation as evidenced by more CD206 and decreased iNOS staining when compared to the control. In addition, WB was performed to quantify protein expression of CD206 and iNOS, and the results showed the same trend with IF staining (Fig. S5). It is possible that PDA and Au NPs synergistically regulate the immune microenvironment by inducing M2 macrophage polarization [26, 40].

**Fig. 4:**
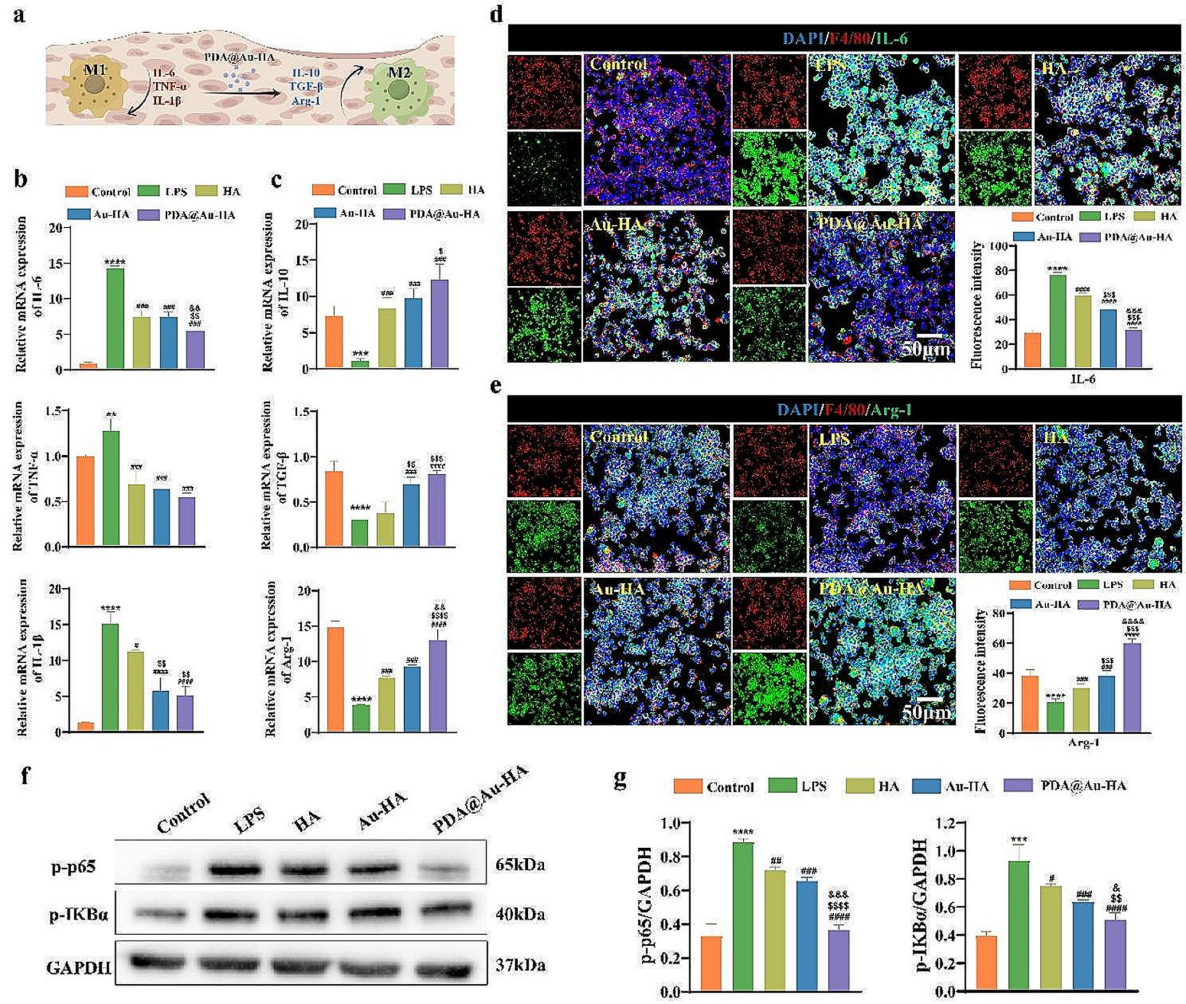
Evaluation of the anti-inflammation function in vitro of PDA@Au-HA. RAW264.7 cells were cultured with indicated groups. (control, LPS, HA, Au-HA and PDA@Au-HA. (**a**) PDA@Au-HA anti-inflammatory pattern diagram was drawn by Figdraw. (**b**) The mRNA levels of pro-inflammatory genes IL-1β, TNF-α, IL-6. (**c**) The mRNA levels of anti-inflammatory genes TGF-β, IL-10, Arg-1. (**d**) IF images of different samples (control, LPS, HA, Au-HA, PDA@Au-HA) stained for IL-6 and (**e**) Arg-1(scale bar:50 μm), and the fluorescence intensity of IL-6 and Arg-1. (**f-g**) The protein levels of p-p65 and p-pIKBα in RAW264.7 treated with HA, Au-HA and PDA@Au-HA NPs for 24 h and corresponding quantitative analysis. (*n* = 3. **p < 0.01, ***p < 0.001 and ****p < 0.0001 indicate significant difference compared to the control group; ^#^*p* < 0.05, ^###^*p* < 0.001 and ^####^*p* < 0.0001 indicate significant difference compared to the LPS group. ^$$^*p* < 0.01, ^$$$^*p* < 0.001 and ^$$$$^*p* < 0.0001indicate significant difference compared to the HA group. ^&^*p* < 0.05, ^&&^*p* < 0.01, ^&&&^*p* < 0.001 and ^&&&&^*p* < 0.0001indicate significant difference compared to the Au-HA group)

Inflammatory cytokines distinctly influence macrophage polarization. LPS significantly stimulated the secretion of M1-associated inflammatory cytokines, and PDA@Au-HA can promote the secretion of M2-associated cytokines **(**Fig. 4a**)**. To further explore the effect of PDA@Au-HA on inflammatory cytokines production in activated macrophage, qRT-PCR analysis was carried out to detect pro-inflammatory markers and anti-inflammatory markers. These results indicated that the gene expression level of IL-1β, TNF-α, and IL-6 upregulated significantly in the LPS group than in the control group, indicating the successful establishment of the inflammatory model. PDA@Au-HA significantly down-regulated the expression level of M1 macrophage-related genes, including TNF-α, IL-6, and IL-1β, whereas up-regulated the expression of pro-healing genes that related to anti-inflammatory M2 macrophages such as Arg-1, TGF-β, and IL-10 compared to the Au-HA and HA groups, indicating that PDA@Au-HA played a vital role in macrophages polarization (Fig. 4b-c). We hypothesized that wound microenvironment modulation and macrophage phenotypic regulation are linked to PDA@Au-HA’s anti-inflammatory impact. For the protein expression, as depicted in Fig. 4d-e, LPS stimulation upregulated intracellular IL-6 protein expression and downregulated Arg-1 protein, the fluorescence intensity of IL-6 was significantly reduced and the Arg-1 intensity was increased in the PDA@Au-HA group compared to Au-HA and HA group. To further explore its potential mechanisms, several key proteins of the NF-κB classical inflammatory pathway were detected to evaluate the possible anti-inflammatory mechanisms of PDA@Au-HA NPs. An increased expression of p-p65 and p-IKBα was observed in RAW 264.7 cells stimulated with LPS for 24 h, thus highlighting the activation of the NF-κB pathway, while PDA@Au-HA significantly reduced the expression of p-p65 and p-IkBα **(**Fig. 4f-g). In summary, PDA@Au-HA could identify the oxidative stress in the wound microenvironment and eliminate the excessive ROS, creating a favorable microenvironment for bone reconstruction during the inflammatory stage of healing.

### The effect of PDA@Au-HA and macrophage-CM on the behavior of MC3T3-E1 in vitro

**Fig. 5:**
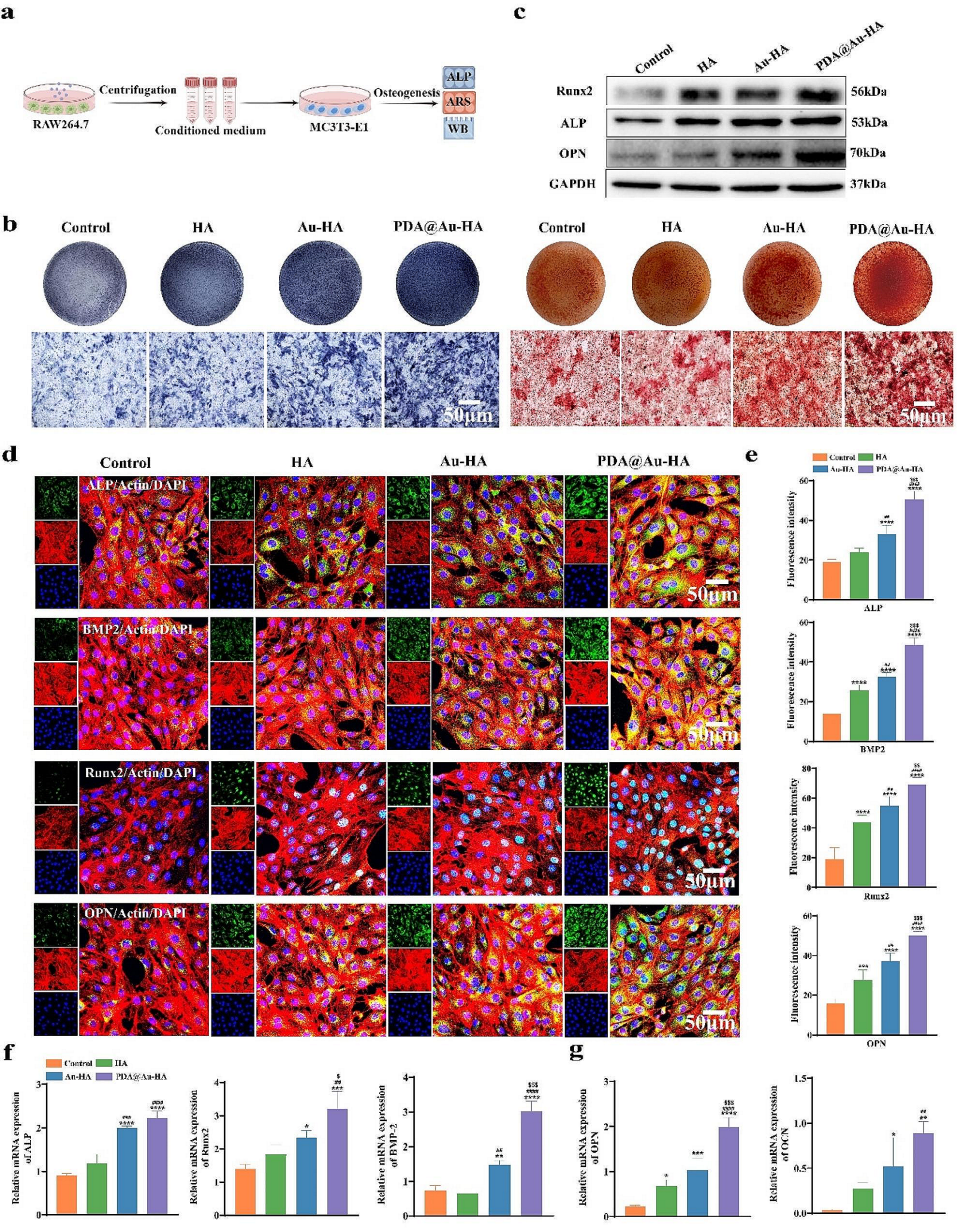
Evaluation of the osteogenetic effect of PDA@Au-HA and macrophage-CM in vitro. MC3T3-E1 cells were cultured with different CM groups (control, HA, Au-HA and PDA@Au-HA). (**a**) Preparation of CM and this was draw n by Figdraw. (**b**) ALP staining of MC3T3-E1 for 7d and ARS staining of MC3T3-E1 for 14 d (scale bar:50 μm); (**c**) WB experiment showing the protein expression including Runx2, ALP and OPN on day 14. (**d**) IF staining results of MC3T3-E1 treated with different CM for 7 days, including ALP, BMP2, Runx2 and OPN, while F-actin and nuclei were stained red by phalloidin and blue by DAPI, respectively (scale bar:50 μm). (**e**) Semiquantitative analysis of the fluorescence intensity of ALP, BMP2, Runx2 and OPN. (**f**) The mRNA levels of ALP, Runx2, and BMP2 in MC3T3-E1 treated with different CM for 7 days. (**g**) The mRNA levels of OPN and OCN in MC3T3-E1 treated with different CM for 14 days. (*n* = 3. *p < 0.05, **p < 0.01, ***p < 0.001 and ****p < 0.0001 indicate significant difference compared to the control group; ^##^*p* < 0.01, ^###^*p* < 0.001 and ^####^*p* < 0.0001 indicate significant difference compared to the HA group. ^$^*p* < 0.05, ^$$^*p* < 0.01 and ^$$$^*p* < 0.001 indicate significant difference compared to the Au-HA group)

First, for 1 day, the macrophages were cultured with various NPs (HA, Au-HA, and PDA@Au-HA). Next, the CM of macrophage was collected. The osteogenic differentiation of MC3T3-E1 cultured with macrophage-CM was evaluated by ALP staining and activity, ARS staining, WB, IF, and RT-PCR **(**Fig. 5a**)**. After 7d of culture, the color of ALP staining of MC3T3-E1 in PDA@Au-HA medium group was darker than that in HA and Au-HA groups (Fig. 5b). Quantitative analysis of ALP activity demonstrated PDA@Au-HA medium group exhibited highest ALP activity (Fig. S6). The extracellular matrix (ECM) mineralization in MC3T3-E1 cultures was assessed by ARS, which represents subsequent osteogenic activity. A similar trend was found in the ARS analysis, the most calcium was found in cells cultured with PDA@Au-HA 14d in its corresponding CM **(**Fig. 5b**).** ALP, Runx2, BMP-2, OCN, and OPN are among the osteoblastic genes that were tested using RT-PCR assays after 7 and 14 days of osteogenic induction to show how PDA@Au-HA media can support MC3T3-E1 osteogenic differentiation at the mRNA level. ALP and Runx2 stimulate osteoblast development and proliferation as well as the maturation of the bone matrix in the early phases of osteogenic differentiation. OCN, a matrix protein released by mature osteoblasts during mineralization, is expressed in the later stages of osteogenic development. The findings revealed that PDA@Au-HA CM encouraged the greatest expression of the above-mentioned five osteogenic-related genes **(**Fig. 5f-g**)**. WB was used to further examine the expression of osteogenesis-related proteins on day 14. As shown in Fig. 5c, the expression of Runx2, ALP and OPN was all promoted by HA, Au-HA and PDA@Au-HA CM, and the PDA@Au-HA exhibited the darkest protein bands. Additionally, relevant quantitative analyses revealed that PDA@Au-HA had the highest expression of each protein (Fig. S7). The IF staining results of MC3T3-E1 treated with HA, Au-HA, PDA@Au-HA medium for 7 days is shown in Fig. 5d. In the fluorescence images ALP, BMP2, Runx2 and OPN were stained green, while the cytoskeleton and nuclei were stained red and blue, respectively. The findings demonstrated that compared to the HA, Au-HA, and control groups, the PDA@Au-HA expression levels of ALP, BMP2, Runx2, and OPN were significantly greater. The protein fluorescence intensity semiquantitative analysis displays a similar pattern **(**Fig. 5e**)**. According to these findings, PDA@Au-HA can considerably increase osteogenic activity in vitro by modifying the immunological milieu mediated by macrophages. The mechanism of the PDA@Au-HA NPs on its regulatory effects on MC3T3-E1 can be summarized into three aspects. On the one hand, PDA contributes to cell adhesion, which may activate cytoskeletal tension through the RhoA/ROCK signaling pathway [41, 42] and promote osteogenic differentiation of MC3T3-E1, the PDA@Au-HA NPs promoted M2 macrophages to secrete osteogenesis-related cytokines, M2 macrophage expression of BMP2 and TGF-β promotes osteogenic differentiation [43]. On the other hand, Au NPs are osteogenic and differentiating, and Au NPs combined with calcium phosphate synergistically promote MC3T3ombined with calcium phosphate synergistically promote MC3T3-E1 osteogenesis [44]. We speculate that Au NPs may target the autophagy-lysosome system to combat inflammatory damage to promote cellular osteogenesis [45].

### PDA@Au-HA NPs promoted angiogenesis in HUVECs cultures in vitro

**Fig. 6:**
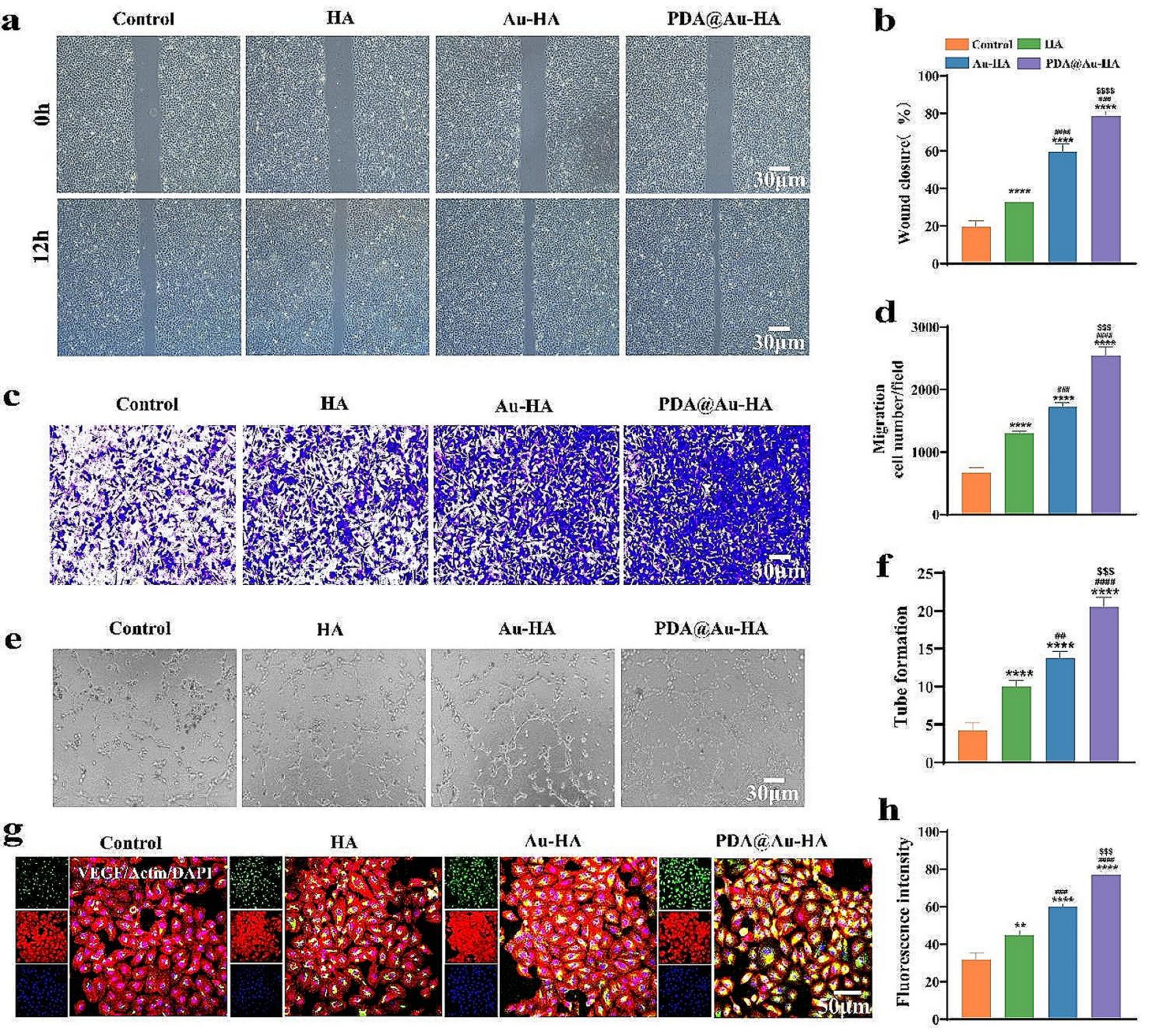
Evaluation of angiogenic capability of PDA@Au-HA in vitro. (**a**) Scratch experiment of HUVECs cultured with indicated groups. (control, HA, Au-HA and PDA@Au-HA) (scale bar:30 μm). (**b**) Quantitative analysis based on wound closure (%) in 12 h. (**c**) The HUVECs were stained with crystal violet to test tissue migration in 24 h (scale bar:30 μm). (**d**) Quantification of migrated HUVECs cells number. (**e**) Leica microscope images of the tube formation assay (scale bar:30 μm). (**f**) Quantitative analysis of the tube formation structure. (**g**) Representative IF images of VEGF (scale bar:50 μm). (**h**) Quantitative analysis the VEGF fluorescence intensity. (*n* = 3. **p < 0.01and ****p < 0.0001 indicate significant difference compared to the control group; ^##^*p* < 0.01, ^###^*p* < 0.001 and ^####^*p* < 0.0001 indicate significant difference compared to the HA group. ^$$$^*p* < 0.001 and ^$$$$^*p* < 0.0001 indicate significant difference compared to the Au-HA group)

It has been established that a successful method for encouraging bone regeneration in the early phases of bone healing is comprehensive revascularization [46, 47]. Therefore, the angiogenic properties of NPs were evaluated using HUVECs by scratch and tube formation tests to mimic the in vivo process of angiogenesis. The photos were taken to record the area of the marginal cells that migrated into the scratches to close the gap. The PDA@Au-HA treated group demonstrated the least blank area and the best cell migration property among the four groups (Au-HA, HA, control groups) (Fig. 6a-b). The PDA@Au-HA-treated group showed a statistically significant rise, according to the quantitative analysis. A Transwell assay was performed by seeding cells in an upper chamber with materials in a lower chamber. After incubation for 24 h, the PDA@Au-HA groups exhibited a greater number of permeabilized cells in the lower region of the transwell insert in comparison to the other three groups **(**Fig. 6c-d**)**. Furthermore, a tube formation assay was performed to replicate angiogenesis in a three-dimensional setting, and the Matrix fundamentally better reflects the ability of vascular endothelial cells to remodel an ECM [46]. The outcome revealed that PDA@Au-HA groups generated more tubular structures **(**Fig. 6e-f**)**. IF staining was conducted to further investigate the expression level of angiogenic-related protein VEGF after culturing HUVECs with PDA@Au-HA. Similar to the scratch wound and tube formation experiments, IF showed a similar trend **(**Fig. 6g-h**)**.

### PDA@Au-HA NPs promote osteogenesis in rat skull defect models

**Fig. 7:**
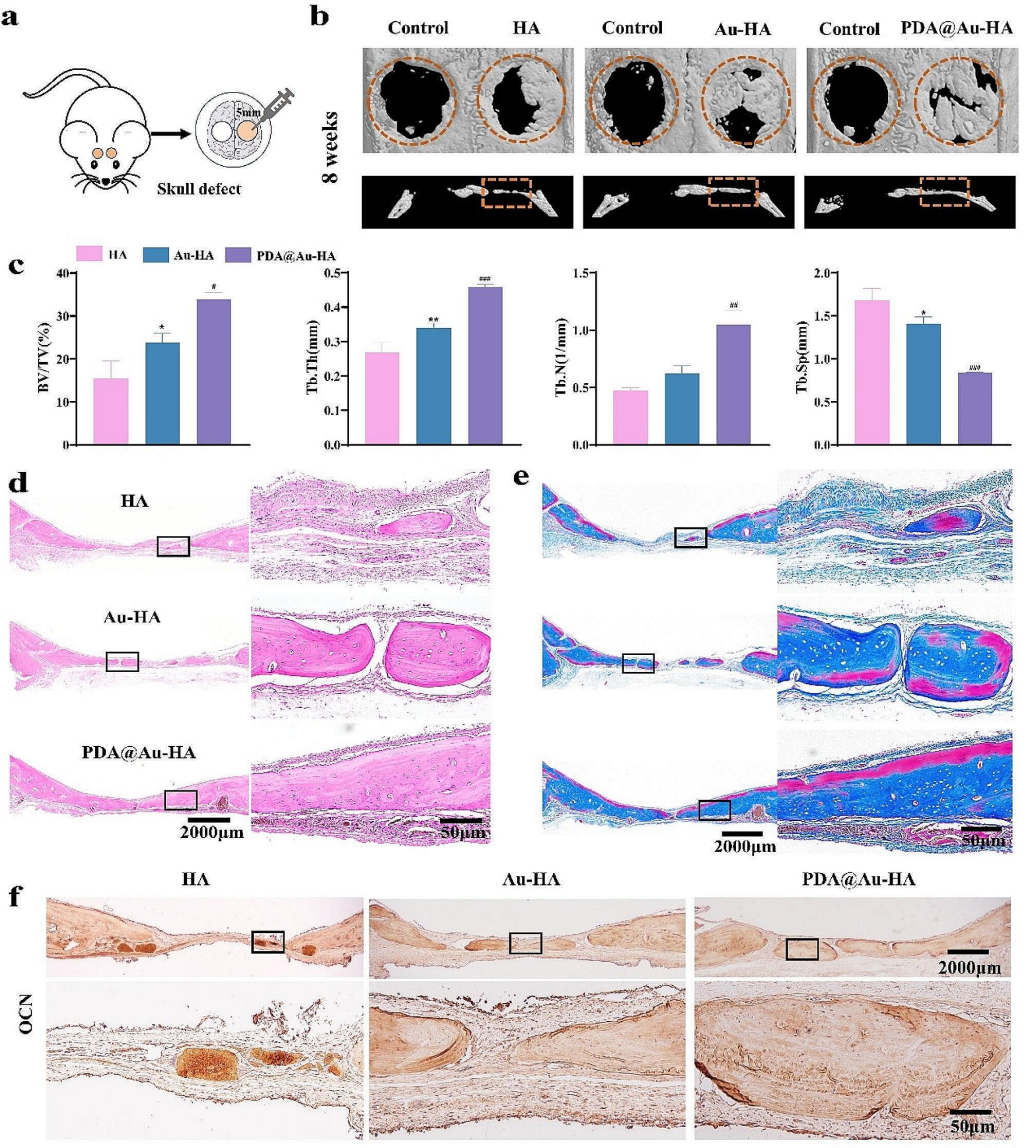
Bone repair performance of PDA@Au-HA NPs in vivo. (**a**) Schematic illustration of the creation of the rat skull defect model. (**b**) Micro-CT reconstructive images of the defects 8 weeks after the surgery. (**c**) BV/TV, Tb.N, Tb.Sp, Tb.Th of rat cranial defects determined by Micro-CT. (**d**) H&E staining images; (**e**) Masson staining images. (**f**) IHC staining of OCN. (scale bar:2000 μm; scale bar:50 μm; *n* = 3. *p < 0.05 and **p < 0.01 indicate significant difference compared to the HA group; ^##^*p* < 0.01 and ^###^*p* < 0.001 indicate significant difference compared to the Au-HA group)

In order to determine the bone regenerative ability of the PDA@Au-HA in vivo. We implanted the different NPs onto bone defects in rats **(**Fig. 7a**)**. Eight weeks after implantation, the rats were sacrificed and cranial bone specimens were collected. First, 3D reconstruction micro-CT images showed that the amount of newly formed bone varied among the four groups compared with the initial size of the defect, represented by a red circle. And the PDA@Au-HA group had the greatest amount of newly formed bone tissue, suggesting that PDA@Au-HA NPs produced better microenvironments for bone regeneration than HA and Au-HA **(**Fig. 7b**)**. In addition, quantitative analysis showed a similar trend with Micro-CT, the bone volume/total volume (BV/TV), trabecular number (Tb·N) and trabecular thickness (Tb·Th) showed that the group treated with PDA@Au-HA NPs exhibited the best bone healing outcomes (Fig. 7c). Micro-CT showed less bone in the control group, so we did not analyze the control group for bone volume and H&E staining. The new bone tissue was then histologically stained using H&E and Masson’s staining. H&E staining mostly indicates the presence of inflammation and new bone. According to the H&E staining results, the PDA@Au-HA group displayed more new bone and more soft tissue aggregates than the HA and Au-HA groups **(**Fig. 7d**)**. Masson’s staining shows where the collagen has been deposited. In the PDA@Au-HA group, collagen deposition was noticeably denser and higher **(**Fig. 7e**).** The IHC results in Fig. 7f further demonstrate that compared to HA and Au-HA, the PDA@Au-HA NPs elevated the expression of OCN, among which PDA@Au-HA NPs showed the most obvious promoting effect. These results again suggest that the multifactorial synergy of PDA@Au-HA NPs create an optimal microenvironment for bone regeneration.

#### Effects of the PDA@Au-HA on skin wounds healing in vivo

**Fig. 8:**
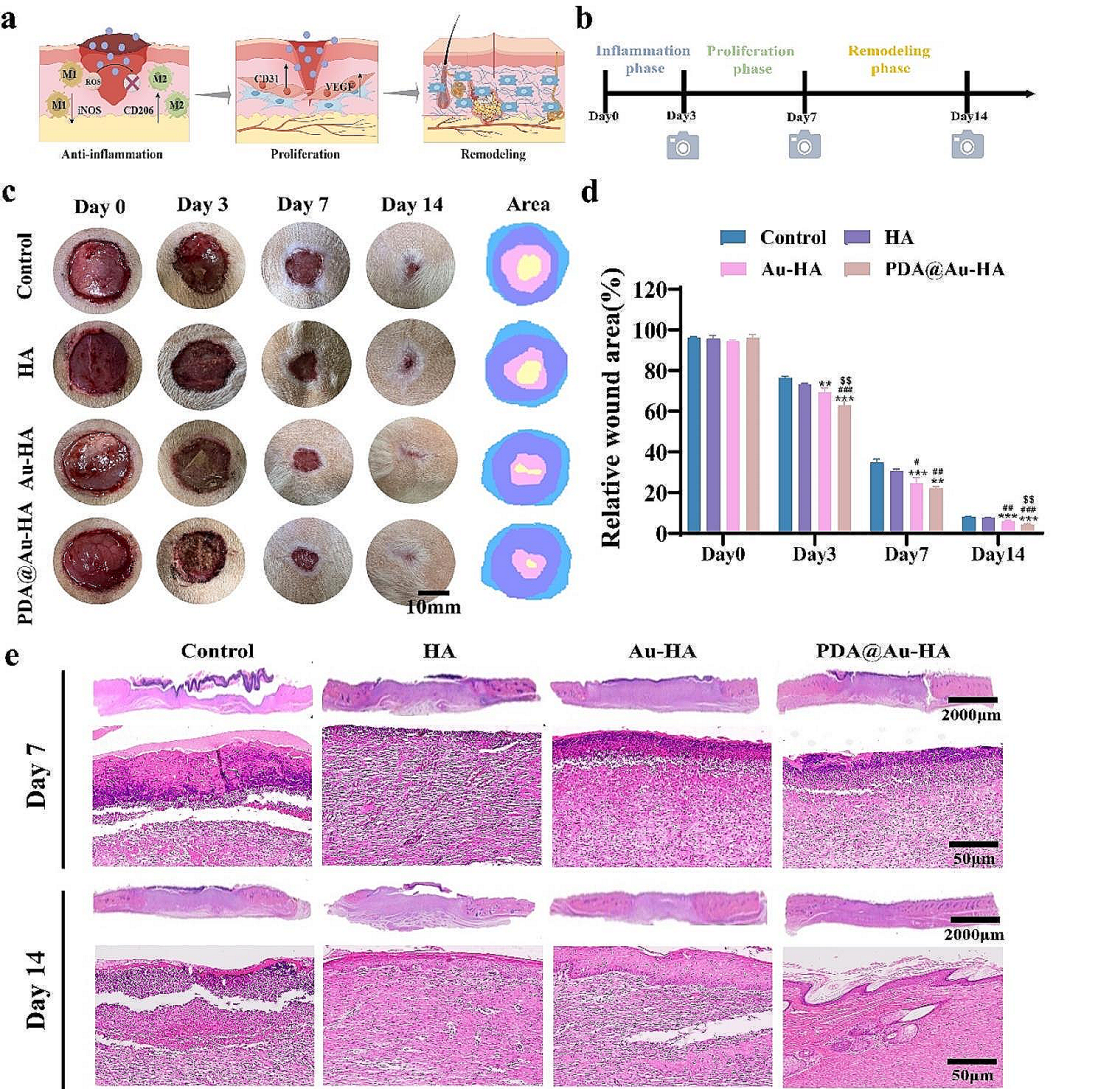
Wound closure analysis in SD rat models. (**a**) Wound healing three-stage model diagram was drawn by Figdraw. (**b**) Flow chart of wound healing experiment. (**c**) Representative digital photographs of wounds on days 0, 3, 7 and 14 (scale bar:10 mm). (**d**) Relative wound area analysis for all the groups on days 0, 3, 7 and 14. (**e**) H&E staining of wound tissues at 7d and 14d. (upper scale bar:2000 μm; lower scale bar:50 μm; *n* = 3. **p < 0.01, and ***p < 0.001 indicate significant difference compared to the control group; ^#^*p* < 0.05, ^##^*p* < 0.01 and ^###^*p* < 0.001 indicate significant difference compared to the HA group; ^$$^*p* < 0.01 indicate significant difference compared to the Au-HA group)

PDA@Au-HA NPs have demonstrated strong biocompatibility, the capacity to promote cell migration and proliferation, and the ability to construct microvascular lumens, according to several in vitro experiments. To further demonstrate that PDA@Au-HA can modulate the immune microenvironment in vivo to promote tissue regenerative repair, we established a rat skin wound defect model. 24 SD rats were used to construct a skin defect model and divided into four groups, with 6 rats in each group (control, HA, Au-HA, PDA@Au-HA). A circular full-flap skin defect with a diameter of 2 cm was created on the dorsal side of each rat, and record wound healing at specific times. Different NPs (200 µg/ml) were applied to the wound site and treated every day. Figure 8a shows a wound healing three-stage model diagram. After different treatments, the wound healing process was monitored (Fig. 8b). As shown in Fig. 8c, we photographed and recorded the wound area changes at several time points (0,3,7,14 day) on the back of the rats. Macroscopically, wound shrinkage of the PDA@Au-HA group was the most obvious and rapid within 14 days compared to the control, HA, and Au-HA group. On day 14, the scar size of wounds in the PDA@Au-HA group was significantly smaller than that in the other group. These results showed that the PDA@Au-HA NPs successfully accelerated wound healing and prevented scar formation in vivo. Quantitative analysis also showed that the PDA@Au-HA-treated group has the highest healing ratios on days 7 and 14 **(**Fig. 8d**)**. Histological analysis of the wound tissues was performed to characterize the regenerative effects of the different NPs, and the skin samples were collected on days 7 and 14. H&E staining results were consistent with the wound healing rate. The H&E staining images **(**Fig. 8e**)** showed that there were still many inflammatory cells in the control, HA, and Au-HA groups at day 7, but less inflammation was observed in the PDA@Au-HA. On day 14, the epithelial layer healing was incomplete in the control group. The epithelial layer of HA and Au-HA group is basically formed. Specifically, significant rete pegs and keratinocyte repair were visible in the PDA@Au-HA-treated group (Fig. 8e**)**. Taken together, the results demonstrate that the PDA@Au-HA has superior wound healing ability compared to the other groups.

**Fig. 9:**
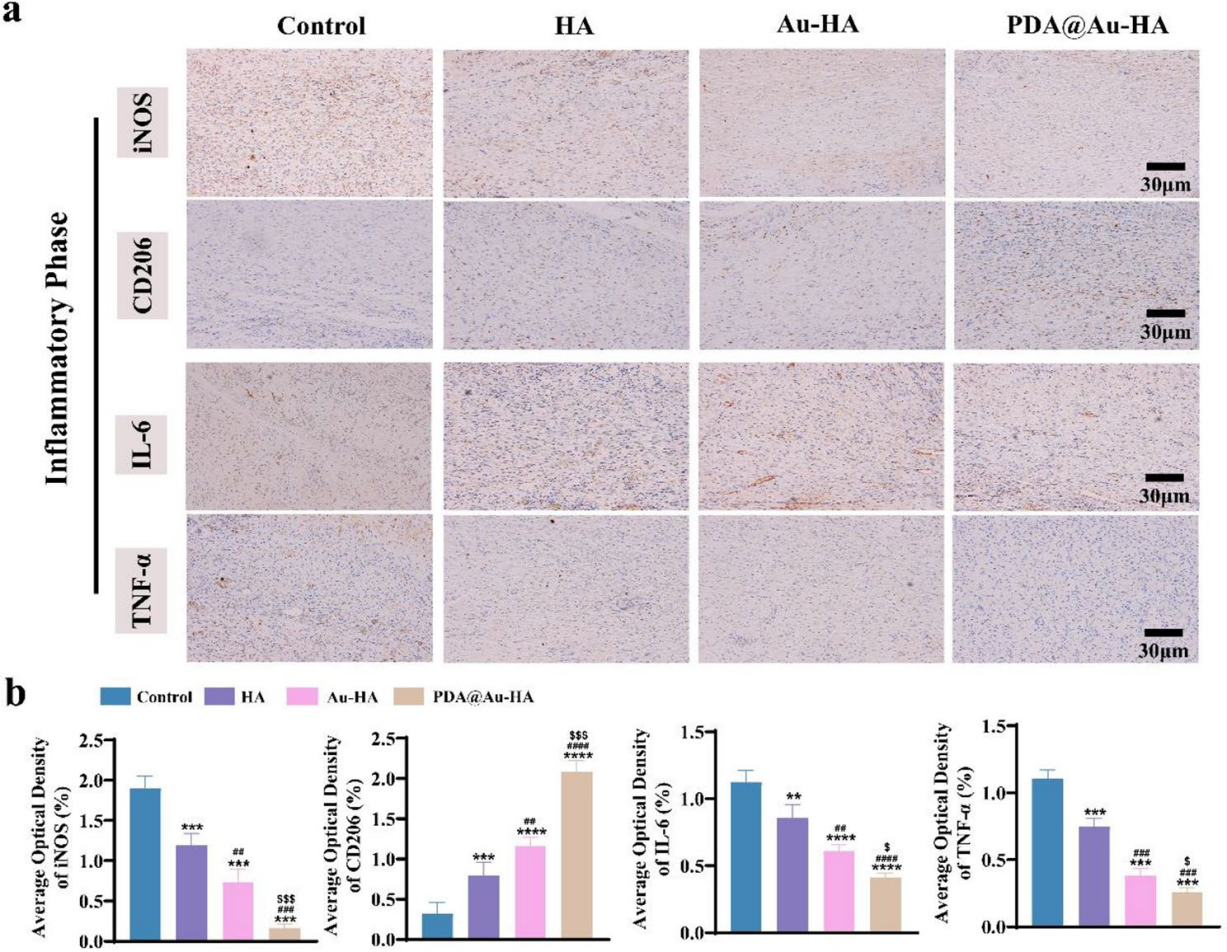
Inflammatory response of different NPs to wounds at an early stage. (**a**) IHC staining of iNOS, CD206, IL-6, and TNF-α on day 3 (scale bar:30 μm). (**b**) Quantitative analysis of iNOS, CD206, IL-6, and TNF-α during the inflammatory phase. (*n* = 3. **p < 0.01, ***p < 0.001 and ****p < 0.0001 indicate significant difference compared to the control group; ^#^*p* < 0.05, ^##^*p* < 0.01, ^###^*p* < 0.001 and ^####^*p* < 0.0001 indicate significant difference compared to the HA group; ^$^*p* < 0.05 and ^$$$^*p* < 0.001 indicate significant difference compared to the Au-HA group)

The three overlapping but separate phases of wound healing are remodeling, proliferation, and inflammation [48]. In physiological wounds, the initial inflammatory phase typically lasts two to three days, but it can last longer in chronic wounds [49]. It is always characterized by infiltration of neutrophils, macrophages, and high levels of pro-inflammatory cytokines. Thus, we analyzed the effects of different NPs on regulating macrophages heterogeneity and inflammatory factor secretion. As shown in Fig. 9a-b, the expression of iNOS (M1) in the PDA@Au-HA-treated wound is significantly lower, while the expression of CD206 (M2) is significantly higher, suggesting that the PDA@Au-HA can convert pro-inflammatory M1 macrophages to anti-inflammatory M2 macrophages. In comparison with the other groups, IL-6 and TNF-α expression was substantially decreased in the PDA@Au-HA groups. This result indicated that the PDA@Au-HA can reduce inflammation by regulating macrophages heterogeneity, increasing the conversion of M1 phenotype macrophages to M2 phenotype, and reducing the accumulation of inflammatory factors.

**Fig. 10:**
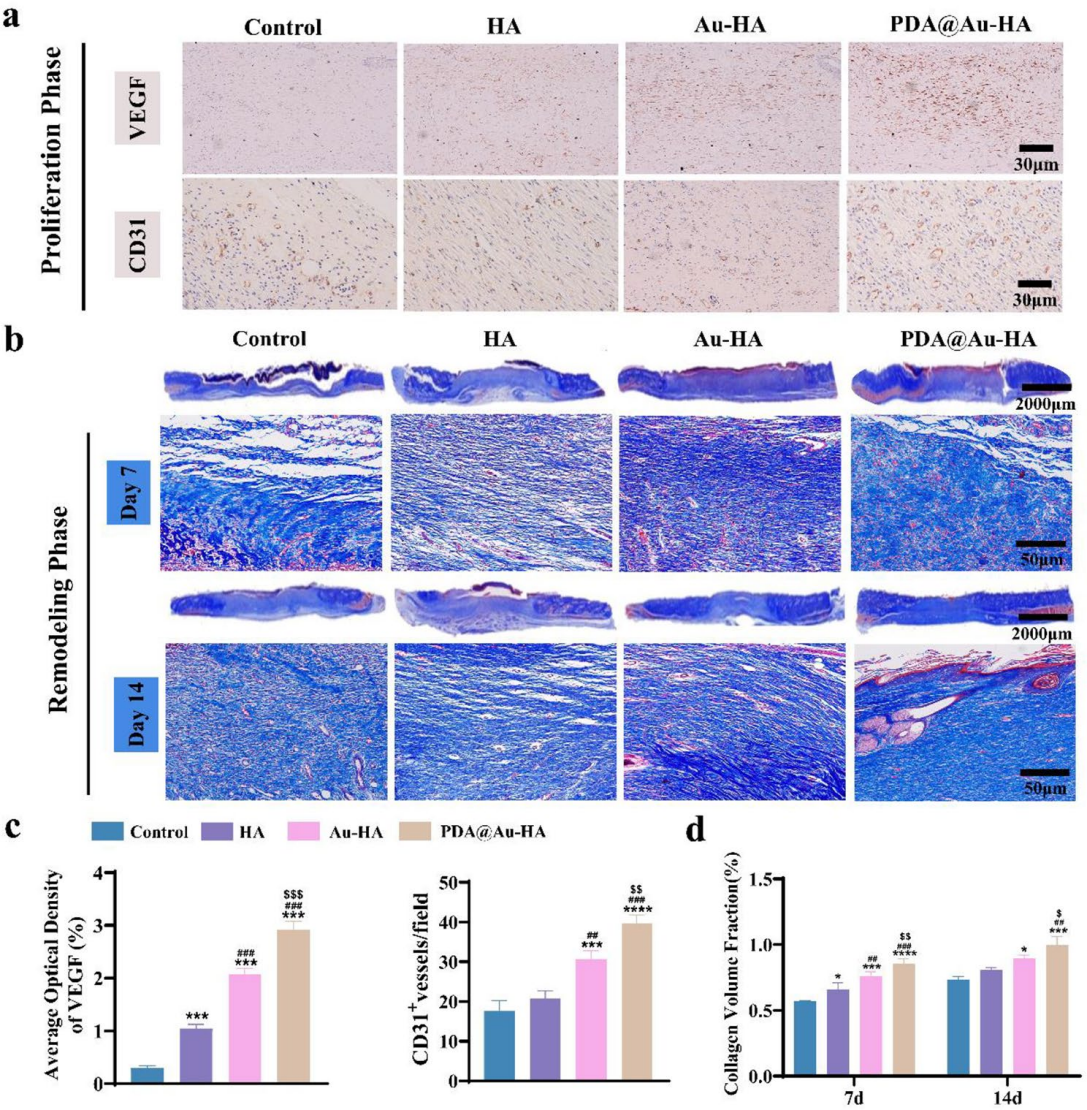
Effect of different NPs on wound repair and reconstruction. (**a**) IHC staining of VEGF and CD31 in the wound area at the proliferation phase (scale bar:30 μm). (**b**) Masson’s trichrome staining at days 7 and 14 (upper scale bar:2000 μm, lower scale bar:50 μm); (**c**) Quantitative analysis of the CD 31 micro-vessel density and VEGF. (**d**) Quantitative analysis collagen volume fraction during the remolding phase. (*n* = 3. *p < 0.05, ***p < 0.001 and ****p < 0.0001 indicate significant difference compared to the control group, ^##^*p* < 0.01 and ^###^*p* < 0.001 indicate significant difference compared to the HA group; ^$^*p* < 0.05, ^$$^*p* < 0.01 and ^$$$^*p* < 0.001 indicate significant difference compared to the Au-HA group)

Neovascularization is essential during the proliferation phase to guarantee that nutrients and oxygen are transported to the wound site to support fibroblast proliferation, collagen synthesis, and re-epithelialization, which speeds up wound healing [50, 51]. In this study, the early angiogenesis during wound healing was assessed by CD31 and VEGF. The PDA@Au-HA group expressed a higher amount of VEGF and CD31, it can promote vascularization and thereby accelerate wound closure compared with other groups **(**Fig. 10a**)**. Specifically, quantification of the positive field for CD31 showed the highest amount of CD31 expression in the PDA@Au-HA group **(**Fig. 10c**)**. Appropriate collagen deposition and remodeling are crucial throughout the remodeling phase to enhance the tissue’s tensile strength and promote healing [52]. Consequently, Masson’s trichrome staining was used to see the collagen that had been produced. At day 14, some crusting is still seen in the control and HA group. Masson staining shows that the PDA@Au-HA treated wounds exhibit better collagen deposition compared to the other groups. Moreover, in the PDA@Au-HA group, there were also a lot of newly formed arteries, neatly arranged collagen fibers, and skin projections like follicles **(**Fig. 10b**)**. And the PDA@Au-HA-treated wounds showed a higher collagen volume fraction (Fig. 10d). All these in vivo animal experiment results demonstrated that the PDA@Au-HA is able to regulate the immune microenvironment at the trauma site, reduced inflammation, promoted angiogenesis, and stimulated re-epithelialization and collagen deposition to accelerate wound healing processes.

So far, although we have had some success, there are some limitations to our study. Firstly, in this study, we explored the effect of different NPs on cell migration in a physiological environment, whereas the effect on cells in an inflammatory environment was not investigated. Secondly, we did not dynamically observe rat cranial regeneration at different time points. Thirdly, we need to establish different animal models to further explore the properties of this NPs and optimize it in the future. Most importantly, we will further explore the mechanistic pathways by which this nanomaterial modulates the immune microenvironment and promotes vascularized bone regeneration.

## Conclusion

In summary, using a strategy inspired by mussels, we successfully constructed a PDA@Au-HA multifunctional nanoplatform for modulating the microenvironment in the wound region and inducing vascularized bone regeneration to accelerate bone reconstruction. The nanocomposites were prepared by simple depositional precipitation. The nanocomposite exhibits many advantages in bone regenerative repair. Firstly, the catechol group on PDA promoted cell adhesion and conferred the nanocomposites with ROS scavenging and anti-inflammatory properties. Secondly, Au and HA play a synergistic role in bone repair. More importantly, PDA@Au-HA nanocomposites regulate the crosstalk between macrophages and osteoblasts to promote bone regeneration by inhibiting M1 polarization and promoting the secretion of osteogenesis-associated factors by M2 macrophages. Therefore, this nanocomposite is a potential bone replacement material with promising clinical translational applications.

### Electronic supplementary material

Below is the link to the electronic supplementary material.

Supplementary Material 1

## Data Availability

The data that support the findings of this study are available from the corresponding author upon reasonable request.

## References

[1] Han C, Guo M, Bai J, Zhao L, Wang L, Song W, Zhang P. Quercetin-loaded nanocomposite microspheres for chronologically promoting bone repair via synergistic immunoregulation and osteogenesis. Mater Design. 2022; 222.

[2] Deschaseaux F, Sensébé L, Heymann D (2009). Mechanisms of bone repair and regeneration. Trends Mol Med.

[3] Arron JR, Choi Y (2000). Bone versus immune system. Nature.

[4] Chen S, Chen X, Geng Z, Su J (2022). The horizon of bone organoid: a perspective on construction and application. Bioact Mater.

[5] Yang G, Liu H, Cui Y, Li J, Zhou X, Wang N, Wu F, Li Y, Liu Y, Jiang X, Zhang S (2021). Bioinspired membrane provides periosteum-mimetic microenvironment for accelerating vascularized bone regeneration. Biomaterials.

[6] Hankenson KD, Dishowitz M, Gray C, Schenker M (2011). Angiogenesis in bone regeneration. Injury.

[7] Ramasamy SK, Kusumbe AP, Schiller M, Zeuschner D, Bixel MG, Milia C, Gamrekelashvili J, Limbourg A, Medvinsky A, Santoro MM, Limbourg FP, Adams RH (2016). Blood flow controls bone vascular function and osteogenesis. Nat Commun.

[8] Mantovani A, Biswas SK, Galdiero MR, Sica A, Locati M (2013). Macrophage plasticity and polarization in tissue repair and remodelling. J Pathol.

[9] Wang Y, Zhang H, Hu Y, Jing Y, Geng Z, Su J. Bone repair biomaterials: a perspective from Immunomodulation. Adv Funct Mater. 2022; 32 (51).

[10] Locati M, Curtale G, Mantovani A, Diversity (2020). Mechanisms, and significance of macrophage plasticity. Annu Rev Pathol.

[11] Tian Y, Li Y, Liu J, Lin Y, Jiao J, Chen B, Wang W, Wu S, Li C (2022). Photothermal therapy with regulated Nrf2/NF-kappaB signaling pathway for treating bacteria-induced periodontitis. Bioact Mater.

[12] Sun X, Gao J, Meng X, Lu X, Zhang L, Chen R (2021). Polarized macrophages in Periodontitis: characteristics, function, and Molecular Signaling. Front Immunol.

[13] Yang B, Pang X, Li Z, Chen Z, Wang Y (2021). Immunomodulation in the treatment of Periodontitis: Progress and perspectives. Front Immunol.

[14] Li Y, Yang L, Hou Y, Zhang Z, Chen M, Wang M, Liu J, Wang J, Zhao Z, Xie C, Lu X (2022). Polydopamine-mediated graphene oxide and nanohydroxyapatite-incorporated conductive scaffold with an immunomodulatory ability accelerates periodontal bone regeneration in diabetes. Bioact Mater.

[15] Ding T, Kang W, Li J, Yu L, Ge S (2021). An in situ tissue engineering scaffold with growth factors combining angiogenesis and osteoimmunomodulatory functions for advanced periodontal bone regeneration. J Nanobiotechnol.

[16] Ma W, Wang W, Liu F, Kong Y, Xia B, Yang H, Zhao H, Wang L, Li K, Li Y, Sang Y, Liu H, Wang X, Qiu J. Osteoinduction-immunomodulation dual-functional calcium nervonate nanoparticles for promoting bone regeneration. Compos Part B: Eng. 2023; 255.

[17] Wang Y, Wang J, Gao R, Liu X, Feng Z, Zhang C, Huang P, Dong A, Kong D, Wang W (2022). Biomimetic glycopeptide hydrogel coated PCL/nHA scaffold for enhanced cranial bone regeneration via macrophage M2 polarization-induced osteo-immunomodulation. Biomaterials.

[18] Cui Y, Li H, Li Y, Mao L (2022). Novel insights into nanomaterials for immunomodulatory bone regeneration. Nanoscale Adv.

[19] Sun Y, Gao Z, Zhang X, Xu Z, Zhang Y, He B, Yang R, Zhang Q, Yang Q, Liu W (2022). 3D-printed, bi-layer, biomimetic artificial periosteum for boosting bone regeneration. Bio-Design Manuf.

[20] Zhou H, Lee J (2011). Nanoscale hydroxyapatite particles for bone tissue engineering. Acta Biomater.

[21] Sathiyavimal S, Vasantharaj S, Lewisoscar F, Pugazhendhi A, Subashkumar R (2019). Biosynthesis and characterization of hydroxyapatite and its composite (hydroxyapatite-gelatin-chitosan-fibrin-bone ash) for bone tissue engineering applications. Int J Biol Macromol.

[22] Qiao M, Tang W, Xu Z, Wu X, Huang W, Zhu Z, Wan Q (2023). Gold nanoparticles: promising biomaterials for osteogenic/adipogenic regulation in bone repair. J Mater Chem B.

[23] Gupta A, Singh S (2022). Multimodal potentials of gold nanoparticles for bone tissue Engineering and Regenerative Medicine: avenues and prospects. Small.

[24] Liang H, Xu X, Feng X, Ma L, Deng X, Wu S, Liu X, Yang C (2019). Gold nanoparticles-loaded hydroxyapatite composites guide osteogenic differentiation of human mesenchymal stem cells through Wnt/beta-catenin signaling pathway. Int J Nanomed.

[25] Cai F, Li S, Huang H, Iqbal J, Wang C, Jiang X (2022). Green synthesis of gold nanoparticles for immune response regulation: mechanisms, applications, and perspectives. J Biomed Mater Res A.

[26] Ni C, Zhou J, Kong N, Bian T, Zhang Y, Huang X, Xiao Y, Yang W, Yan F (2019). Gold nanoparticles modulate the crosstalk between macrophages and periodontal ligament cells for periodontitis treatment. Biomaterials.

[27] Liang H, Jin C, Ma L, Feng X, Deng X, Wu S, Liu X, Yang C (2019). Accelerated bone regeneration by Gold-Nanoparticle-Loaded Mesoporous Silica through stimulating Immunomodulation. ACS Appl Mater Interfaces.

[28] Li H, Yin D, Li W, Tang Q, Zou L, Peng Q (2021). Polydopamine-based nanomaterials and their potentials in advanced drug delivery and therapy. Colloids Surf B Biointerfaces.

[29] Gong J, Ye C, Ran J, Xiong X, Fang X, Zhou X, Yi Y, Lu X, Wang J, Xie C, Liu J. Polydopamine-mediated Immunomodulatory Patch for Diabetic Periodontal tissue regeneration assisted by Metformin-ZIF system. ACS Nano. 2023. 10.1021/acsnano.3c02407 37578444

[30] Bao X, Zhao J, Sun J, Hu M, Yang X (2018). Polydopamine nanoparticles as efficient scavengers for reactive oxygen species in Periodontal Disease. ACS Nano.

[31] Cheng W, Zeng X, Chen H, Li Z, Zeng W, Mei L, Zhao Y (2019). Versatile polydopamine platforms: synthesis and promising applications for Surface Modification and Advanced Nanomedicine. ACS Nano.

[32] Lee H, Dellatore SM, Miller WM, Messersmith PB (2007). Mussel-inspired surface chemistry for multifunctional coatings. Sci (New York NY).

[33] Yang Y, Liang Y, Chen J, Duan X, Guo B (2022). Mussel-inspired adhesive antioxidant antibacterial hemostatic composite hydrogel wound dressing via photo-polymerization for infected skin wound healing. Bioact Mater.

[34] Schanze KS, Lee H, Messersmith PB (2018). Ten years of polydopamine: current status and future directions. ACS Appl Mater Interfaces.

[35] Liu X, Man HC (2017). Laser fabrication of Ag-HA nanocomposites on Ti6Al4V implant for enhancing bioactivity and antibacterial capability. Mater Sci Eng C Mater Biol Appl.

[36] Zhang J, He X, Yu S, Zhu J, Wang H, Tian Z, Zhu S, Cui Z (2021). A novel dental adhesive containing Ag/polydopamine-modified HA fillers with both antibacterial and mineralization properties. J Dent.

[37] Treccani L, Yvonne Klein T, Meder F, Pardun K, Rezwan K (2013). Functionalized ceramics for biomedical, biotechnological and environmental applications. Acta Biomater.

[38] Sui L, Wang J, Xiao Z, Yang Y, Yang Z, Ai K (2020). ROS-Scavenging nanomaterials to treat Periodontitis. Front Chem.

[39] Shapouri-Moghaddam A, Mohammadian S, Vazini H, Taghadosi M, Esmaeili SA, Mardani F, Seifi B, Mohammadi A, Afshari JT, Sahebkar A (2018). Macrophage plasticity, polarization, and function in health and disease. J Cell Physiol.

[40] Bai X, Chen D, Dai Y, Liang S, Song B, Guo J, Dai B, Zhang D, Feng L (2021). Bone formation recovery with gold nanoparticle-induced M2 macrophage polarization in mice. Nanomedicine.

[41] Wang YK, Yu X, Cohen DM, Wozniak MA, Yang MT, Gao L, Eyckmans J, Chen CS (2012). Bone morphogenetic protein-2-induced signaling and osteogenesis is regulated by cell shape, RhoA/ROCK, and cytoskeletal tension. Stem Cells Dev.

[42] Kim J, Kim HD, Park J, Lee ES, Kim E, Lee SS, Yang JK, Lee YS, Hwang NS (2018). Enhanced osteogenic commitment of murine mesenchymal stem cells on graphene oxide substrate. Biomater Res.

[43] Qiao W, Xie H, Fang J, Shen J, Li W, Shen D, Wu J, Wu S, Liu X, Zheng Y, Cheung KMC (2021). Yeung K W K. Sequential activation of heterogeneous macrophage phenotypes is essential for biomaterials-induced bone regeneration. Biomaterials.

[44] Zhang Y, Wang P, Mao H, Zhang Y, Zheng L, Yu P, Guo Z, Li L, Jiang Q. PEGylated gold nanoparticles promote osteogenic differentiation in in vitro and in vivo systems. Mater Design. 2021; 197.

[45] Yin Y, Tian BM, Li X, Yu YC, Deng DK, Sun LJ, Qu HL, Wu RX, Xu XY, Sun HH, An Y, He XT, Chen FM (2022). Gold nanoparticles targeting the autophagy-lysosome system to combat the inflammation-compromised osteogenic potential of periodontal ligament stem cells: from mechanism to therapy. Biomaterials.

[46] Li Y, Zhu J, Zhang X, Li Y, Zhang S, Yang L, Li R, Wan Q, Pei X, Chen J, Wang J (2023). Drug-delivery nanoplatform with synergistic regulation of angiogenesis-osteogenesis coupling for promoting vascularized bone regeneration. ACS Appl Mater Interfaces.

[47] Zhang X, Chen JY, Pei X, Li YH, Feng H, He ZH, Xie WJ, Pei XB, Zhu Z, Wan QB, Wang J (2023). One-Pot Facile Encapsulation of Dimethyloxallyl Glycine by Nanoscale Zeolitic Imidazolate Frameworks-8 for enhancing vascularized bone regeneration. Adv Healthc Mater.

[48] V Falanga (2005). Wound healing and its impairment in the diabetic foot. Lancet (London England).

[49] Gurtner GC, Werner S, Barrandon Y, Longaker MT (2008). Wound repair and regeneration. Nature.

[50] Wang M, Huang X, Zheng H, Tang Y, Zeng K, Shao L, Li L (2021). Nanomaterials applied in wound healing: mechanisms, limitations and perspectives. J Control Release.

[51] Okonkwo UA, Dipietro LA. Diabetes and Wound Angiogenesis. Int J Mol Sci. 2017; 18 (7). 10.3390/ijms18071419 PMC5535911 28671607

[52] Yang M, Zhang J, Shi W, Zhang J, Tao C (2022). Recent advances in metal-organic frameworks and their composites for the phototherapy of skin wounds. J Mater Chem B.

