# Supplementary material for: Mussel-inspired immunomodulatory and osteoinductive dual-functional hydroxyapatite nanoplatform for promoting bone regeneration
Source: J Nanobiotechnology. 2024 Jun 8;22:320. doi: 10.1186/s12951-024-02593-3 (PMC11162024; doi:10.1186/s12951-024-02593-3)
Supplement: Supplementary file 1 — Supplementary Material 1 [file 12951_2024_2593_MOESM1_ESM.docx]

**Supporting Information**

**Mussel-inspired immunomodulatory and osteoinductive dual-functional hydroxyapatite nanoplatform for promoting bone regeneration**

Danlei Qin^1,2,3^, Yifan Zhao^1,2^, Rui Cheng^4^, Yingyu Liu^1,2^, Susu Guo^1,2^, Lingxiang Sun^1,2^, Yanqin Guo^5^, Fengxiang Hao^1,2^, Bin Zhao^1,2*^.

^1^ Shanxi Medical University School and Hospital of Stomatology, Taiyuan, 030001, Shanxi, China

^2^ Shanxi Province Key Laboratory of Oral Diseases Prevention and New Materials, Taiyuan, 030001, Shanxi, China

^3^ Department of Medical Imaging, Shanxi Medical University, Taiyuan 030001, Shanxi, China

^4^ Department of Endocrinology, the Second Hospital of Shanxi Medical University, 382 Wuyi Road, Taiyuan 030001, Shanxi, China

^5^ Department of Ultrasound, the Second Hospital of Shanxi Medical University, 382 Wuyi Road, Taiyuan 030001, Shanxi, China

^*^ **Corresponding Authors:**

^*^ **Bin Zhao:** Shanxi Medical University School and Hospital of Stomatology, Shanxi Province Key Laboratory of Oral Diseases Prevention and New Materials, Shanxi Medical University, Taiyuan, 030001, China

**E-mail**: sxmu0688@126.com


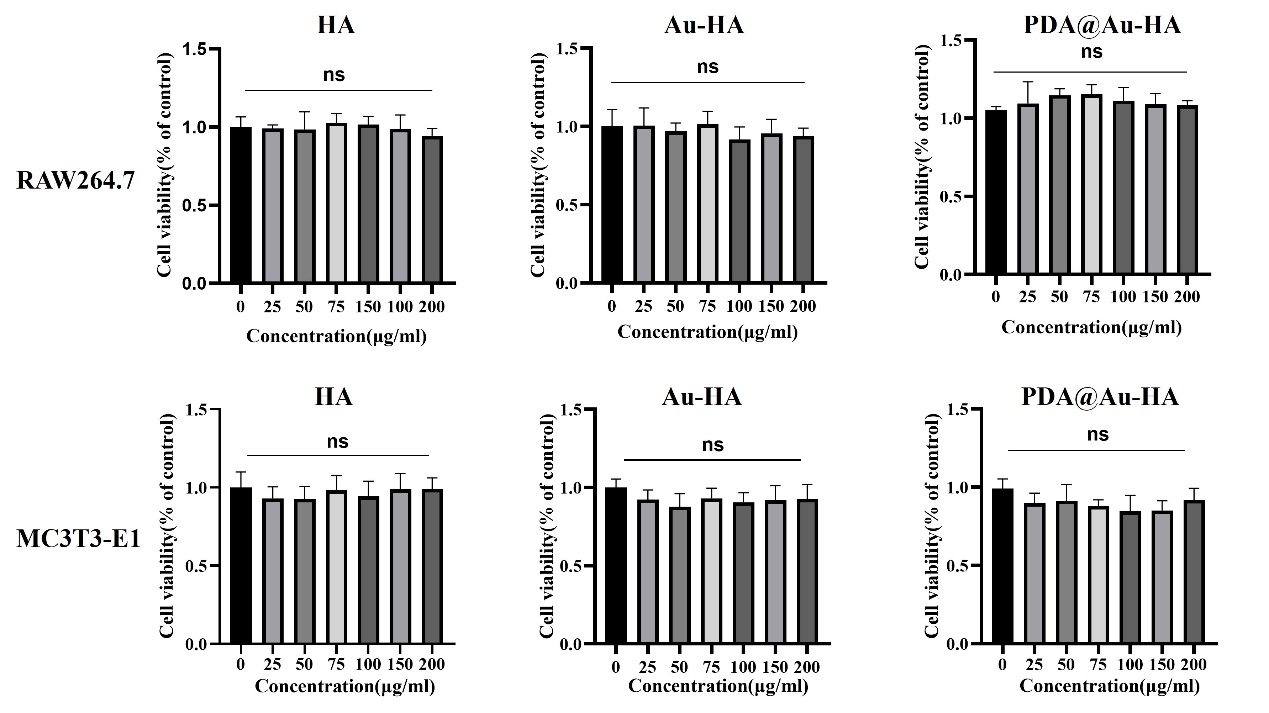
 **Supplementary 1:** The cell viability of RAW 264.7 and MC3T3-E1 treated with different concentrations of HA, Au-HA and PDA@Au-HA NPs for 24 h.
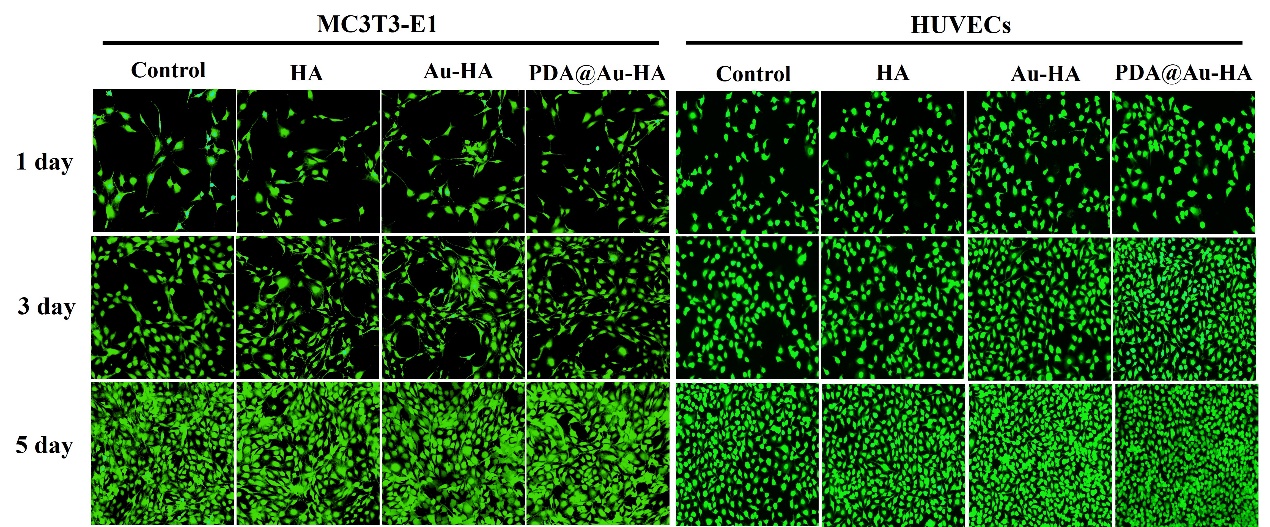


**Supplementary 2:** Live/dead fluorescence images of MC3T3-E1 and HUVECs after incubation with HA, Au-HA and PDA@Au-HA NPs for 1,3,5 days.
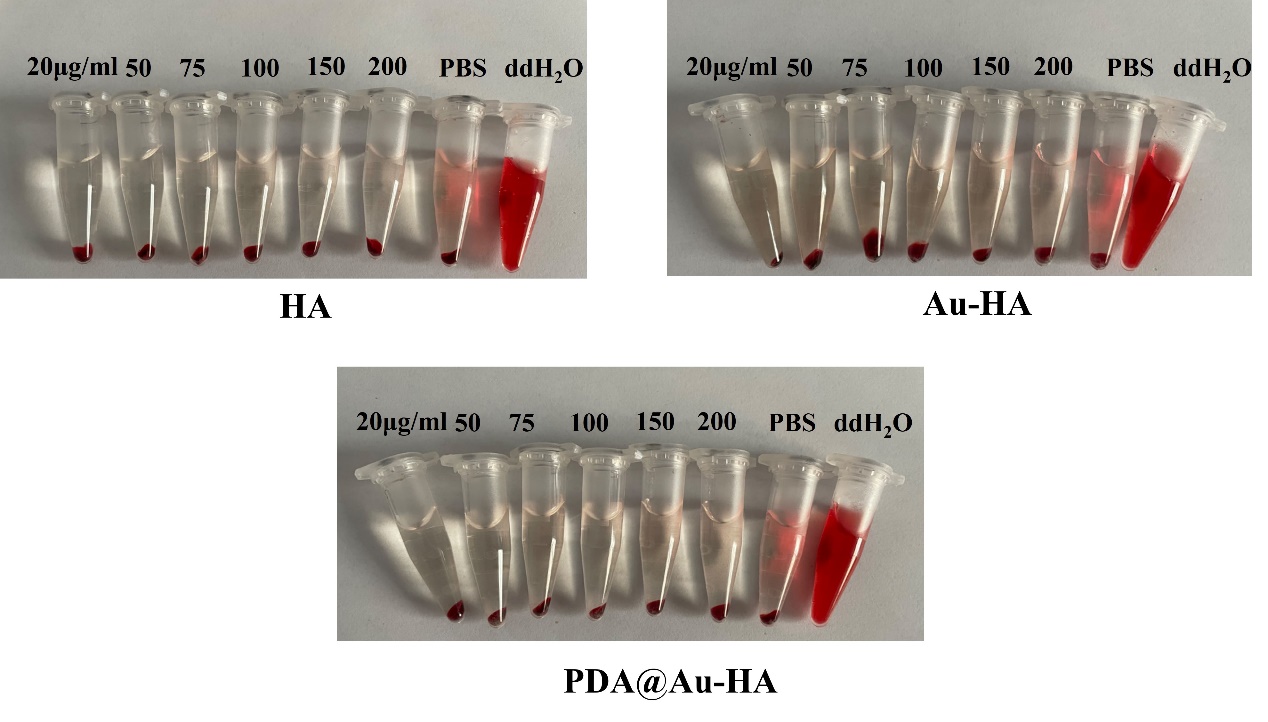


**Supplementary 3:** Image of hemolysis evaluation of different concentration HA, Au-HA and PDA@Au-HA NPs.


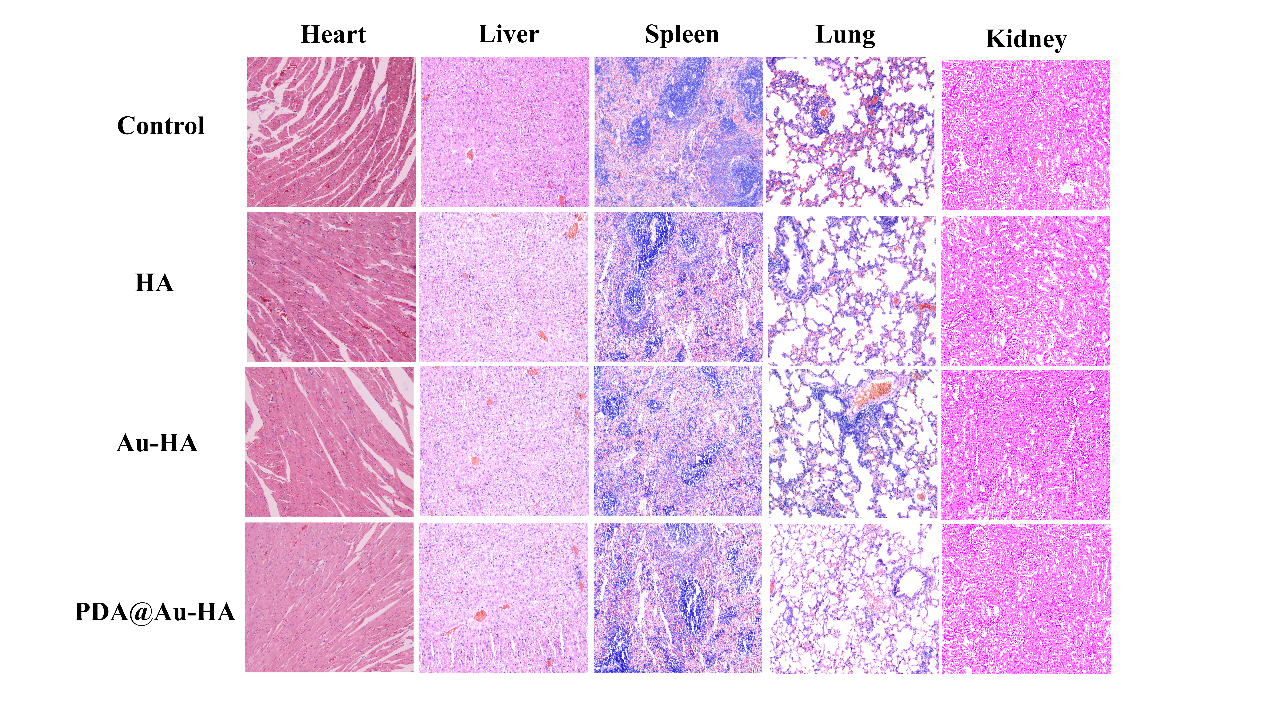


**Supplementary 4:** Biosafety evaluation of in vivo. H&E staining of major organs (heart, liver, spleen, lung, and kidney) in different groups.


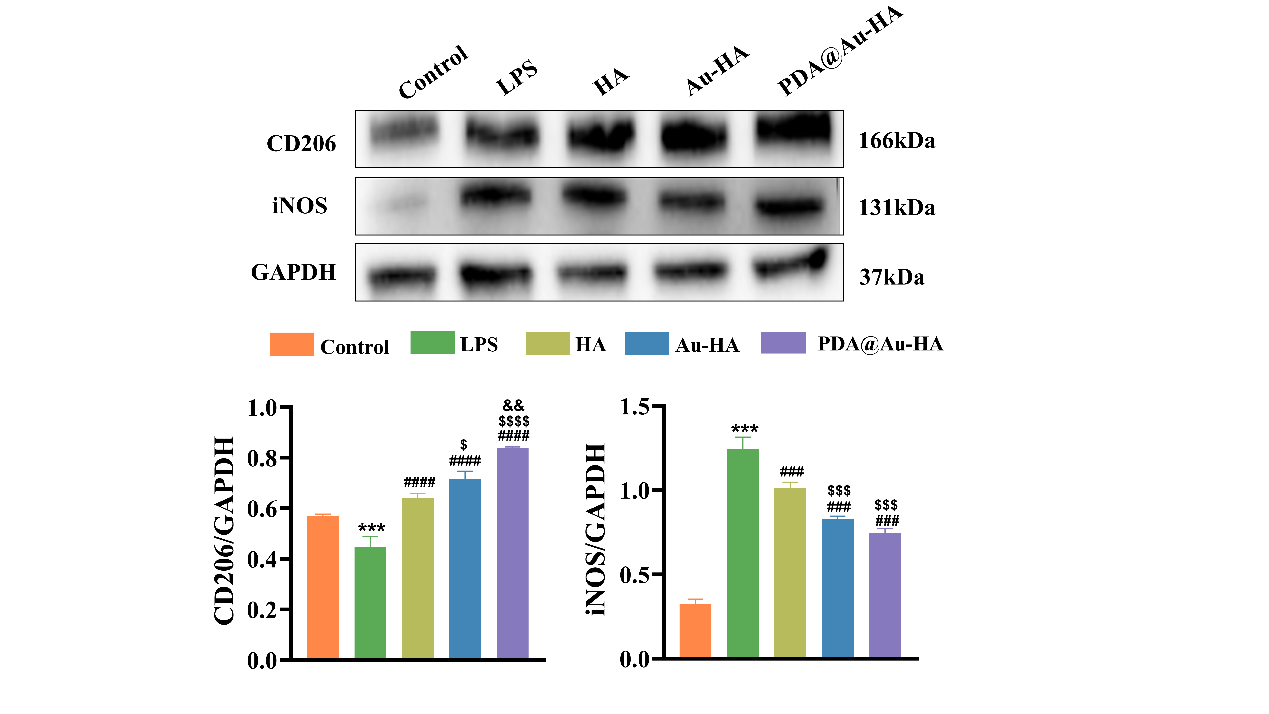


**Supplementary 5:** The protein levels of CD206 and iNOS in RAW264.7 treated with HA, Au-HA and PDA@Au-HA NPs for 24h and corresponding quantitative analyses.

(n=3. ***p < 0.001 indicate significant difference compared to the control group; ^###^p < 0.001 and ^####^p < 0.0001 indicate significant difference compared to the LPS group. ^$^p < 0.05, ^$$$^p < 0.001 and ^$$$$^p < 0.0001 indicate significant difference compared to the HA group. ^&&^p < 0.01 indicate significant difference compared to the Au-HA group).


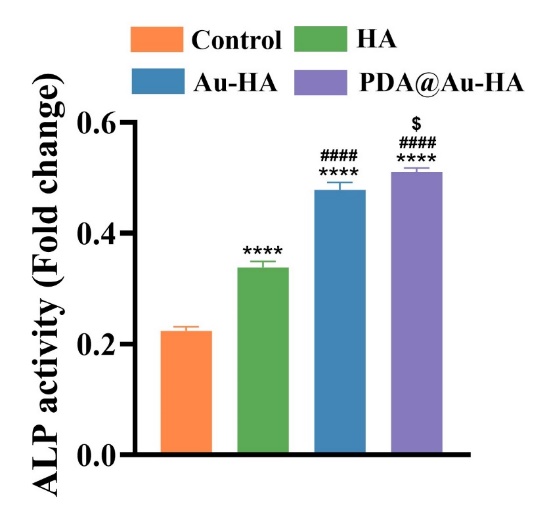


**Supplementary 6:** The ALP activity the quantitative results. (n=3. ****p < 0.0001 indicate significant difference compared to the control group; ^####^p < 0.0001 indicate significant difference compared to the HA group. ^$^p < 0.05 indicate significant difference compared to the Au-HA group).
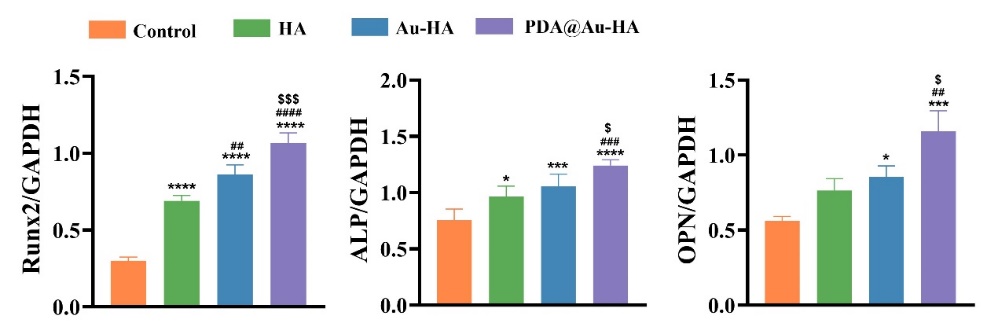


**Supplementary 7:** The quantitative analyses of Runx2, ALP and OPN in MC3T3-E1 treated with HA, Au-HA and PDA@Au-HA NPs for 7 days. (n=3. *p < 0.05, ***p < 0.001 and ****p < 0.0001 indicate significant difference compared to the control group; ^##^p < 0.01, ^###^p < 0.001 and ^####^p < 0.0001 indicate significant difference compared to the HA group. ^$^p < 0.05 and ^$$$^p < 0.001 indicate significant difference compared to the Au-HA group).
